# The Oriented Emergence of Axons from Retinal Ganglion Cells Is Directed by Laminin Contact In Vivo

**DOI:** 10.1016/j.neuron.2011.03.013

**Published:** 2011-04-28

**Authors:** Owen Randlett, Lucia Poggi, Flavio R. Zolessi, William A. Harris

**Affiliations:** 1 Department of Physiology, Development and Neuroscience, University of Cambridge, Cambridge CB2 3DY, UK; 2 Centre for Organismal Studies (COS), Heidelberg, Im Neuenheimer Feld 230, 69120 Heidelberg, Germany; 3 Seccion Biologia Celular, Departamento de Biologia Celular y Molecular, Facultad de Ciencias, Universidad de la Republica, 27101 Montevidee, Uruguay

## Abstract

How the site of axon emergence is specified during neural development is not understood. Previous studies disagree on the relative importance of intrinsic and extrinsic mechanisms. The axons of retinal ganglion cells (RGCs) emerge basally in vivo, yet because RGCs develop from polarized neuroepithelial cells within a polarized environment, disentangling intrinsic and extrinsic influences is a challenge. We use time-lapse imaging to demonstrate that Laminin acting directly on RGCs is necessary and sufficient to orient axon emergence in vivo. Laminin contact with the basal processes of newborn RGCs prevents the cells from entering a stochastic Stage 2 phase, directs the rapid accumulation of the early axonal marker Kif5c560-YFP, and leads to the formation of axonal growth cones. These results suggest that contact-mediated cues may be critical for the site of axon emergence and account for the differences in cellular behavior observed in vitro and in vivo.

**Video Abstract:**

## Highlights

► Axon emergence from RGCs is stochastic in vitro, but is highly directed in vivo ► Laminin is necessary for in vivo behavior and prevents in vitro-like behavior ► Contact with Laminin is sufficient to transform a neurite to an axon in vivo ► Laminin contact directs microtubule changes, leading to axon formation

## Introduction

The extension of axons and dendrites from the cell body marks the dramatic morphological polarization of the typical neuron. This morphology is critical because it is tightly coupled to neuronal network function, where electrical information is picked up in the dendrites and transmitted down the axon. The first step in the generation of neuronal networks, therefore, is the efficient coordination of neuronal polarization. This can also be thought of as the first step in axono/dendritic guidance. Thus, how this orientation decision is regulated, and how the appropriate axis is selected from the myriad of possibilities offered by a 3D tissue, is an important question in developmental neurobiology, yet little is known about how this happens within the embryonic nervous system.

In the late 1980s it was discovered that isolated hippocampal neurons plated on homogeneous substrates first undergo a period of randomly oriented explorations, but then project a single axon and multiple dendrites in the absence of any polarizing extracellular cues (Dotti et al., 1988). These neurons progress through a staged series of behaviors, including a prolonged multipolar phase, known as Stage 2, where dynamic neurites are extended and retracted in various orientations from the cell body. Morphological symmetry is broken in Stage 3, when one neurite grows much longer than the others and develops into the axon (Craig and Banker, 1994). This remarkable cellular behavior of neurons in tissue culture has allowed investigators to identify many factors involved in the extension of a single axon from a set of more or less equivalent neurites (Arimura and Kaibuchi, 2007; Barnes and Polleux, 2009; Tahirovic and Bradke, 2009), and to formulate a general model of cytoskeletal regulation, which lies at the heart of neuronal polarization. In this model, localized actin destabilization in the preaxonal neurite leads to increased microtubule penetration and stabilization in this neurite, which as a result, makes it grow faster than the other neurites and so become the axon (Bradke and Dotti, 1999; Witte et al., 2008). Once selected, the axon upregulates cAMP. This directs the other neurites to become dendrites, which upregulate cGMP. This reciprocal cAMP/cGMP regulation acts as a symmetry-breaking positive feedback loop, ensuring that only a single axon is formed (Shelly et al., 2010).

Strikingly, classes of neurons develop along preferred aligned orientations in vivo, rather than at the random orientations chosen in culture. For example, all retinal ganglion cells (RGCs) send out their axons from the basal part of the cell body (Hinds and Hinds, 1974). What orients axon emergence in vivo? Neurons in the brain derive from highly polarized neuroepithelial cells, with distinct apical and basolateral domains (Randlett et al., 2010). Many cell cycles in advance of any neuronal differentiation, these cells already exhibit polarized behaviors, such as cell divisions only at the apical surface and apically directed movements of the nucleus prior to mitosis during interkinetic nuclear migration (Norden et al., 2009). Also, at the division preceding the neuron's birth, intracellular factors such as the apical complex, the centrosome, and the Golgi apparatus may become localized to one pole of the cell, leading to an intrinsic cellular polarity (Calderon de Anda et al., 2005, 2008).

The model of intrinsically regulated axon emergence meshes well with some studies in cultured hippocampal neurons, where the position of the apical complex and centrosome seems capable of influencing the nearest neurite to become an axon (Calderon de Anda et al., 2005; Shi et al., 2003). Whether the position of apical complex components and the centrosome actually specifies the position of the axon in vivo is, however, controversial. In mice, the initiation of the apically directed axon in cortical pyramidal neurons correlates with the reorientation of the centrosome to a position apical to the nucleus (Calderon de Anda et al., 2010). In *Drosophila*, however, apical complex components and the centrosome appear to be completely dispensable for normally oriented neuronal polarization (Basto et al., 2006; Rolls and Doe, 2004). In zebrafish RGCs, the apical complex and the centrosome are localized apically while the axon emerges basally (Zolessi et al., 2006). Therefore, a clear consensus on the role of intrinsic factors in the generation of oriented axon emergence has not yet been reached.

This led us to examine the polarized environment in which the differentiating neurons reside. Developing neurons in vivo live in an environment that is far from homogeneous because there are extracellular biases along the apico-basal axis. This polarity, we previously argued (Zolessi et al., 2006), could serve to direct the site of axon genesis in vivo. Is it the case that external cues acting directly upon polarizing neurons result in axon emergence toward or away from the stimulus? In support of this idea, neuron polarization in vitro can be directed by asymmetric presentation of Netrin 1, BDNF, TGF-β, cAMP/cGFP, or Sema3a, or by contact with cell adhesion or extracellular matrix molecules (Esch et al., 1999; Gupta et al., 2010; Mai et al., 2009; Ménager et al., 2004; Polleux et al., 1998; Shelly et al., 2007). There is also some in vivo evidence for the importance of extracellular cues directing neuronal polarization in *C. elegans*, where HSN neurons require Netrin/Unc-6 signaling to orient axon extension, and disruptions in Wnt signaling result in inversions in the polarity of PLM and ALM neurons (Adler et al., 2006; Hilliard and Bargmann, 2006; Prasad and Clark, 2006).

Evidence for the importance of extracellular cues in vertebrate neuronal polarization has been more challenging to establish. Recent studies combining in vitro experiments and in vivo electroporation techniques in mice found that the type II TGF-β receptor and LKB1 are required for neuronal polarization in the cortex, and localized BDNF can direct neuronal polarization in vitro through LKB1 phosphorylation. This led to the hypothesis that gradients of TGF-β and/or BDNF could be orienting neuronal polarization in the cortex (Shelly et al., 2007; Yi et al., 2010). However, neurons with disruptions in these genes and elsewhere often fail to put out axons at all, leaving the question of the initial orientation of axons unresolved (Barnes et al., 2007; Calderon de Anda et al., 2010; de la Torre-Ubieta et al., 2010; Kishi et al., 2005; Shelly et al., 2007; Yi et al., 2010).

To investigate whether an extracellular cue does influence the orientation of axonogenesis in vivo, we make use of RGCs in the zebrafish retina. We can image these cells at short time intervals at subcellular resolution from genesis through polarization and axon extension, within a living embryo (Poggi et al., 2005; Zolessi et al., 2006). RGCs are born at the apical surface of the retina, and translocate their cell body toward the basal surface, where the ganglion cell layer will develop. As the apical process detaches from the apical surface of the retina, the axon extends directly from the basal surface of the RGC, showing no prolonged, multipolar, Stage 2 behavior. In *nok* zebrafish mutants, in which the transmembrane protein Pals-1/Stardust is defective, the pigment epithelium is disrupted and newly generated RGCs sometimes come in contact with the basal lamina of the pigment epithelium (Bruch's membrane) instead of the basal lamina of the neural retina. When they do so, they polarize upside-down, suggesting that the basal lamina is responsible for RGC polarity (Zolessi et al., 2006). Here we demonstrate that RGC polarization toward the basal lamina requires the presence of an extracellular cue, Laminin 1 (Lam1). In the absence of Laminin α1 (Lamα1), RGCs exhibit Stage 2 behavior and mispolarization. Contact of newborn RGC processes with Lam1 either in vitro or in vivo is sufficient to cause the specific accumulation of Kif5c560-YFP, a marker of axonal microtubules, followed by axon extension. Thus, in the normal retina, basally localized Lam1 directs the normal orientation of RGC axon extension in vivo.

## Results

### RGC Axon Extension Is Preceded by Directed Kif5c560-YFP Accumulation in the Tip of the Basal Process In Vivo

Live imaging in zebrafish demonstrated that axons extend directly from the most basal portions of RGCs in vivo (Zolessi et al., 2006). This previous study, however, was limited by the unavailability of an intracellular marker of axonogenesis. The earliest reported marker for definitive axon commitment during hippocampal neuron polarization is the constitutively active motor domain of Kinesin 1 fused to YFP, Kif5c560-YFP. This construct selectively accumulates in axons, directed by biochemical differences in axonal microtubules; perhaps reflecting stabilized microtubules (Hammond et al., 2010; Jacobson et al., 2006; Konishi and Setou, 2009). During Stage 2, Kif5c560-YFP displays a remarkably dynamic behavior, where YFP signal accumulates in just one (or sometimes a few) neurites, but this accumulation is only transient, passing from one neurite through the cell body to another neurite (Jacobson et al., 2006). As the neuron progresses to Stage 3 and the axon is selected, Kif5c560-YFP accumulates specifically and permanently in the tip of the preaxonal process, and remains there during axon extension (Jacobson et al., 2006). Thus, the oscillatory behavior provides a visual readout of the uncommitted Stage 2 phase, and the cessation of this oscillation and stable Kif5c560 accumulation in one neurite marks axonal commitment.

**Figure 1:**
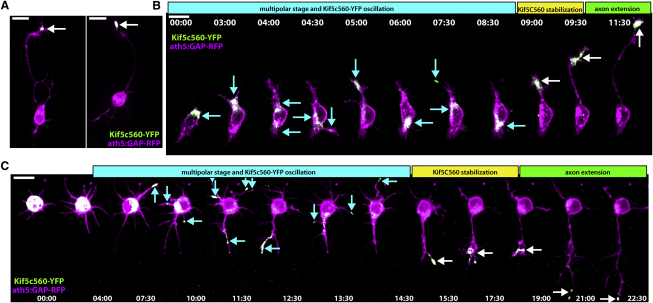
Kif5c560-YFP Marks the Axon of Cultured RGCs and Oscillates during Stage 2 Retinal ganglion cells (RGCs) were dissociated from *ath5:GAP-RFP* embryos injected with Kif5c560-YFP mRNA at the one-cell stage and allowed to polarize in vitro. (A) Bright Kif5c560-YFP was seen in the growth cone of the long axon projecting from polarized RGCs (arrow). (B and C) Shortly after plating, RGCs exhibit Kif5c560-YFP oscillations in different areas of the cell body and individual neurites, typical of Stage 2 behavior (arrows, cyan phase). The construct eventually stabilizes in a single neurite (white arrows, yellow phase), and this neurite extends to form the axon (green phase). Figure is related to Movie S1. Time is shown in hr:min; scale bars = 10 μM.

To see whether this marker of axonogenesis behaves in the same manner in zebrafish RGCs, we performed time-lapse imaging of *ath5:GAP-RFP* transgenic embryos injected with Kif5c560-YFP RNA at the one-cell stage to label RGCs (Poggi et al., 2005; Zolessi et al., 2006). At 30 hours post-fertilization (hpf), the approximate onset of RGC genesis, eyes were dissected and dissociated to obtain isolated cells. After a 12–15 hr incubation at 28.5°C, many RGCs had extended long axons, with bright Kif5c560-YFP signal accumulation within their growth cones (Figure 1A). This confirms that the rat construct maintains its abilities to recognize axonal microtubules and to accumulate in the axonal growth cones of zebrafish RGCs. To assess the dynamics of Kif5c560-YFP during polarization, *ath5:GAP-RFP*-positive neurons were imaged for 12–20 hr beginning at 1–2 hr after plating. Consistent with what was reported for cultured hippocampal neurons, the YFP signal in multipolar RGCs demonstrated an oscillatory behavior, where signal accumulation was seen traveling between different neurites and areas within the cell (Figures 1B and 1C, Movie S1, available online). The YFP signal eventually stabilized in a single neurite, which extended to form the axon. From these data we conclude that this construct behaves identically in cultured RGCs and hippocampal neurons, and cultured RGCs progress through a bona fide Stage 2 phase during polarization.

**Figure 2:**
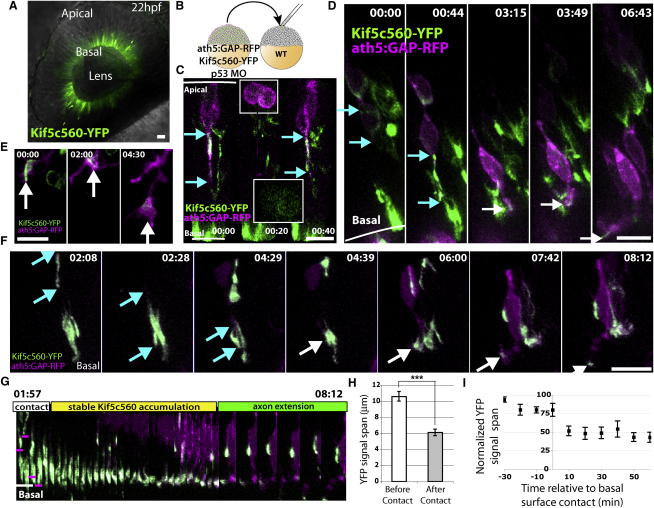
Kif5c560-YFP Marks the Axon in RGCs In Vivo, and Accumulates in a Highly Directed Manner at the Tip of the Basal Process Prior to Axon Extension (A) Injection of Kif5c560-YFP mRNA at the one-cell stage results in expression in all retinal cells, and demonstrates that Kif5c560 accumulates basally in neuroepithelial cells. Image is a single confocal slice. (B) Transplantation scheme used to create mosaic embryos expressing *ath5:GAP-RFP* and Kif5c560-YFP in a subset of RGCs. (C) Kif5c560-YFP signal accumulation (marked by cyan arrows) is confined to the basal process of *ath5*-expressing neuroepithelial cells (t = 0:00) and to young postmitotic neuroblasts (t = 0:40), but is diffuse during mitosis (t = 0:20, inset). (D) In very young RGCs with low *ath5:GAP-RFP* signal, Kif5c560-YFP signal is seen in the cell body (t = 0:00, YFP span marked by cyan arrows), but quickly moves to the basal process (t = 0:44), to the tip of the basal process (t = 3:15, white arrows), and finally to the extending axonal growth cone (t = 6:43). (E) A higher-magnification example showing an RGC growth cone (arrows) navigating within the retina and demonstrating strong and specific Kif5c560-YFP accumulation. (F) Time-lapse image demonstrating that Kif5c560-YFP signal initially spans a large portion of the basal process (span marked by cyan arrows), but upon contact with the basal surface, YFP signal accumulates specifically at the tip of the basal process (white arrows), and finally in the extending axon. (G) Kymograph of the cell shown in (F), demonstrating that immediately after the RGC basal process (marked by magenta line) contacts the basal surface of the retina (white line), the Kif5c560-YFP signal accumulates specifically at the tip of the basal process before the axon extends. (H) Measuring the length of Kif5c560-YFP signal span demonstrates a significant decrease in length after basal surface contact (mean ± SEM, p < 0.0001, Mann-Whitney test, n = 7 cells from three embryos). (I) Plotting the YFP signal span normalized to the longest observed length for each cell (mean ± SEM) demonstrates that the specific accumulation of YFP occurs immediately after basal surface contact (t = 0). Frames are taken from Movie S2. Time is shown in hr:min; scale bars = 10 μM.

We next analyzed how this construct behaves in RGCs polarizing in vivo. Injection of in vitro synthesized Kif5c560-YFP RNA resulted in homogeneous expression in all cells. It was immediately evident that Kif5c560-YFP accumulates basally in the retinal neuroepithelium, even before neurogenesis begins, resulting in a ring of YFP signal surrounding the lens (Figure 2A). To assess the localization and dynamics of Kif5c560-YFP at the single-cell level, we used a transplantation approach to create mosaic embryos (Figure 2B). *ath5:GAP-RFP* transgenic embryos were used because all RGCs are brightly labeled through *ath5*-regulated fluorescent protein expression, and RGCs can be imaged from before their birth through polarization and axon extension (Poggi et al., 2005). These embryos were injected with Kif5c560-YFP RNA and P53 morpholino at the one-cell stage, and blastomeres were transplanted into unlabeled host embryos, generating mosaic embryos, where individual cell behaviors could be tracked by time-lapse confocal microscopy. Using this strategy it was apparent that the Kif5c560-YFP construct accumulates basally in all neuroepithelial cells during interphase, being mostly confined to the basal processes. During mitosis and cytokinesis, however, diffuse labeling in the cell body was seen (Figure 2C). The lack of spindle microtubule labeling during mitosis is consistent with the idea that Kif5c560 recognizes stabilized microtubules, and will not label the dynamic spindle microtubules.

Consistent with the in vitro data, imaging of RGC axons extending within the eye demonstrated that Kif5c560-YFP accumulates specifically in the growth cone (Figures 2D–2F, Movie S2). Unlike what happens in vitro, however, we found that Kif5c560-YFP accumulation was highly directed in polarizing RGCs in vivo. At the end of the final mitosis marking the birth of RGCs, when RFP signal begins to increase in neonatal RGCs, Kif5c560-YFP is still mainly in the cell body (Figure 2D). Very soon after this, however, a Kif5c560-YFP-positive basal process extends from the cell body. The YFP signal spans a large portion of the re-extending basal process at this time (red arrowheads, Figures 2D and 2F, Movie S2). At the point when the basal process appears to contact the basal surface of the retina, the YFP signal accumulates very specifically in the tip of the process (Figure 2G). This was quantified by measuring the span of the YFP signal in the basal process at the time points immediately preceding and following the time when the tip of the basal process had extended to reach its final, and most basal, position. Pooling the data from three time points before contact and three time points after contact, a significant change in the span of the YFP signal within the basal process is observed (Figure 2H), where the length decreases from 10.6 ± 0.6 μm (mean ± SEM) before contact, to 6.1 ± 0.4 μm after contact (p < 0.0001: Mann-Whitney test, n = 7 cells from three embryos). Normalizing the measured lengths to the longest length observed for each cell, and centering the data on basal surface contact (t = 0), the trend of decreasing Kif5c560-YFP signal length immediately following basal surface contact is apparent (Figure 2I). This specific accumulation remains until a Kif5c560-YFP-positive growth cone sprouts from the cell and extends toward the optic nerve head. The YFP signal remains accumulated in the growth cone throughout this extension, and is not visible in the remainder of the cell.

### Laminin at the ILM Is Required for Directed Axon Extension

**Figure 3:**
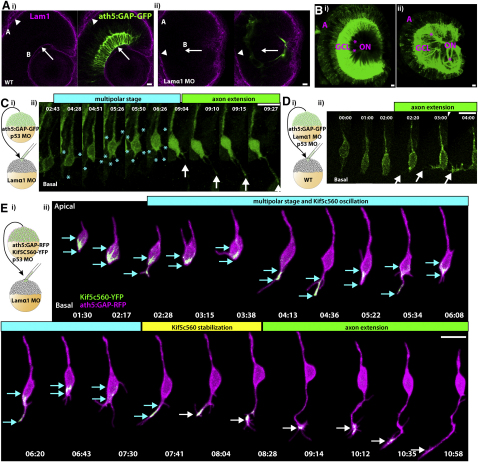
Lamα1 Is Necessary for Directed RGC Polarization (Ai) Immunofluorescent staining of *ath5:GAP-GFP* embryos with polyclonal rabbit anti-Lam1 antibody reveals strong staining at the basal lamina lining the basal surface of the retina, or ILM (B, arrow), as well as at the basal lamina of the RPE, or Bruch's membrane (A, arrowhead). (Aii) Injection of an antisense morpholino targeted against Lamα1 results in efficient loss of Lam1 staining at the basal surface (arrow), while Lam1 staining at Bruch's membrane remains. Images are of a single confocal slice. (B) Confocal reconstruction from a WT 3 dpf retina (Bi) demonstrating the highly ordered nature of the ganglion cell layer (GCL) and the RGC axon fascicles (^∗^) collecting to form the optic nerve (ON). (Bii) After *lamα1* morpholino injection, the GCL is disorganized, as are the axon fascicles, which meander through the retina before colleting to form the ON. (Ci) Mosaic embryos with WT *ath5:GAP-GFP*-labeled RGCs in a *lamα1* morphant environment were analyzed by time-lapse confocal microscopy beginning at approximately 35 hpf. (Cii) RGCs in this environment progress through a transient multipolar phase (marked by [^∗^], cyan phase) before projecting an axon (arrow, green phase). (Di) Mosaic embryos with *lamα1* morphant, *ath5:GAP-GFP*-labeled RGCs in a WT environment were analyzed by time-lapse confocal microscopy. (Dii) Morphant RGCs behave normally in this environment, and axons project directly from the basal surface of the cell (marked by arrowheads). (Ei) Mosaic embryos with WT *ath5:GAP-GFP*-labeled, Kif5c560-YFP-expressing RGCs in a *lamα1* morphant environment were analyzed by time-lapse confocal microscopy. (Eii) In this context, Kif5c560-YFP signal accumulation (marked by cyan arrows) oscillates between the cell body and transient neurites (cyan phase) before stably accumulating in a single neurite (marked by white arrows, yellow phase) that extends to form the axon (green phase). Note that the individual confocal z-slices were cropped to remove signal not associated with the cell. A reconstruction of the uncropped frames is shown in Movie S5. Frames are taken from Movies S3, S4, and S5. Time is shown in hr:min; scale bars = 10 μM.

Because the specific accumulation of Kif5c560-YFP at the tip of the basal process correlates, both in time and in space, with RGCs contacting the basal surface of the retina, we hypothesized that an extracellular cue localized to this region plays a role in this event. The extracellular matrix component Lam1 is a heterotrimer consisting of three subunits (α1, β1, and γ1), and contact with Lam1 is known to be able to polarize neurons in vitro and promote axon growth in RGCs (Esch et al., 1999; Ménager et al., 2004). Moreover, it has been shown that zebrafish embryos lacking the Lamα1 subunit display severe axon guidance defects in multiple neuronal types, including RGCs (Paulus and Halloran, 2006). Using a polyclonal antibody raised against Lam1, strong staining is seen at the basal lamina lining the surfaces of the zebrafish retina (Figure 3Ai) (Lee and Gross, 2007), making it a strong candidate for directing RGC polarization.

To test the necessity for Lamα1 in the normal polarization of RGCs in vivo, we injected a previously described *lamα1* morpholino (Pollard et al., 2006) into *ath5:GAP-GFP* transgenic embryos. Morpholino injections generally resulted in a complete loss of the Lam1 staining at the ILM (Figure 3Aii). Strong Lam1 staining remained at Bruch's membrane at the apical retinal surface, indicating that other α chains could be compensating for the loss of Lamα1 in this region. However, because the RPE acts as a physical barrier between Bruch's membrane and retinal neurons, for our purposes we can assume that the Lamα1-deficient retina is devoid of any accessible Lam1. When fixed at 3 dpf and imaged by confocal microscopy (Figure 3B), morphants showed an apically displaced RGC layer, and instead of the highly ordered axon fascicles, RGC axons in Lamα1 morphants demonstrated severe intra-retinal disorganization. Axons bundles in morphant retinas often took meandering paths through the retinal neuroepithelium prior to collecting at the optic disk to exit the eye.

This axonal disorganization phenotype could be due to axon misguidance, or to problems with neuronal polarization. To differentiate between these two possibilities, we performed time-lapse imaging experiments. Blastomeres were transplanted from *ath5:GAP-GFP* transgenic embryos to Lamα1 morpholino-injected host embryos, resulting in mosaically labeled WT RGCs in a Lamα1-deficient retina (Figure 3C, Movie S3). In this environment, RGCs were observed to progress through a prolonged multipolar phase, where many short neurites were extended from the cell prior to axon extension. Axon extension was often misoriented, projecting from regions of the cell body other than the most basal point. In contrast, when blastomeres were transplanted from *ath5:GAP-GFP* transgenic embryos injected with Lamα1 morpholino into WT host retinas, RGCs polarized normally (Figure 3D, Movie S4). The multipolar stage was not seen, and axons extended directly from the basal surface of the RGC, confirming that the Lamα1 morphant phenotype is non-cell-autonomous. To quantify this effect, we measured the time spent in a multipolar state. The time elapsed between the extension of the first observable dynamic/unstable neurite and the extension of the stable process that became the axon was measured. If the first process that extended became the axon, then Δt = 0 min. When transplanted into a Lamα1 morphant environment, RGCs spent 169 ± 4 min in a multipolar state (mean ± SEM, n = 10 cells from six embryos, where one cell had Δt = 0), while Lamα1 morphant cells transplanted into the WT environment extended a stable axon after a significantly shorter time, 37 ± 2 min (n = 14 cells from seven embryos, where five cells had Δt = 0: p = 0.0028, Mann-Whitney test). Therefore, in the absence of environmental Laminin, RGCs lose their directed polarization behavior, and progress through a multipolar stage, where multiple short neurites are extended from the cell body.

We next used Kif5c560-YFP to visualize the intracellular polarization behavior in vivo when RGCs lack environmental Lam1. We transplanted cells from *ath5:GAP-RFP* transgenic embryos coinjected with Kif5c560-YFP mRNA into *lamα1* morphant host embryos (Figure 3E, Movie S5). Time-lapse microscopy demonstrated that, similar to RGCs in WT retinas, Kif5c560-YFP is localized to the cell body as *ath5:GAP-RFP* expression begins to increase. As RGCs progressed through the multipolar phase, Kif5c560-YFP accumulated in some transient neurites, but this accumulation was not stable and the signal moved back into the cell body upon process retraction. This led to an oscillation of YFP signal accumulation between the cell body and different short neurites typical of Stage 2 neurons. Kif5c560-YFP eventually stably accumulated in a single neurite, which did not retract, and extended as a growth-cone-tipped axon. Therefore, with respect to the Kif5c560-YFP marker, RGCs polarizing in retinas lacking Lam1 behave more similarly to cultured neurons than they do to RGCs polarizing in WT retinas.

### RGCs Polarizing In Vitro and in Lamα1 Morphant Retinas Show Dynamic Centrosomal Movement

Centrosomal localization has been suggested to be important for neuronal polarization in some neurons (Calderon de Anda et al., 2008, 2010; Zmuda and Rivas, 1998), but not in others (Basto et al., 2006; Seetapun and Odde, 2010). In zebrafish retinal neuroepithelial cells, the centrosome is localized to the tip of the apical process. Live imaging in zebrafish demonstrated that this apical centrosome localization is maintained during RGC axon extension in vivo (Zolessi et al., 2006).

**Figure 4:**
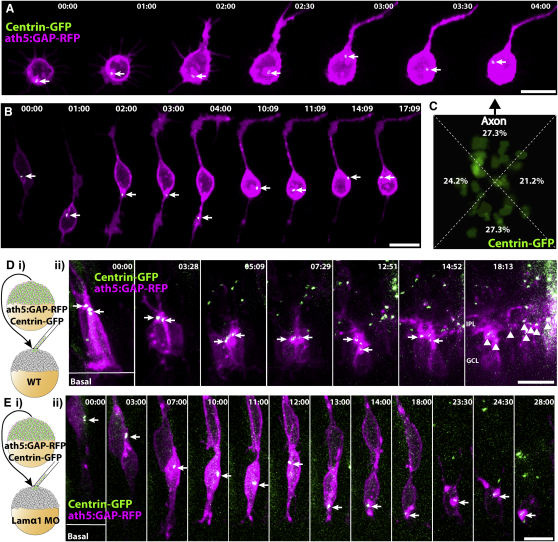
Centrosomes Are Static and Apically Localized in RGCs Polarizing in WT Retinas, but Mislocalized and Dynamic in RGCs Polarizing In Vitro and in *lamα1* Morphants (A and B) RGCs from *ath5:GAP-RFP/Centrin-GFP* transgenic embryos were dissociated and imaged during polarization in vitro. Centrosomes (marked by arrow) are not stably localized, but instead move dynamically within the cell body and even within Stage 2 neurites. (C) Overlay of the centrosomes from 33 polarized RGCs in vitro showing their position in reference to the base of the axon (arrow, *ath5:GAP-RFP* channel not shown). Centroid analysis demonstrates that centrosome location is not biased to any specific quadrant of the RGC cell body (p = 0.9536: Chi square test, n = 33 cells). (Di) Mosaic embryos with WT *ath5:GAP-RFP/Centrin-GFP*-labeled RGCs in a WT environment were analyzed by time-lapse confocal microscopy. (Dii) Centrosomes (marked by arrows) remain apically positioned in RGCs up until dendrite formation and IPL stratification (marked by arrowheads, t = 18:13). (Ei) Mosaic embryos with WT *ath5:GAP-RFP/Centrin-GFP*-labeled RGCs in a *lamα1* morphant environment were analyzed by time-lapse confocal microscopy. (Eii) The centrosome (marked by arrow) is initially localized apically (t < 07:00), but becomes mislocalized during RGC polarization, and moves dynamically within the cell body. Frames are taken from Movies S6 and S7. Time is shown in hr:min; scale bars = 10 μM.

To examine the role of the centrosome in RGC polarization further, we first dissociated RGCs from *ath5:GAP-RFP/Centrin-GFP* transgenic embryos and imaged them during axon extension (Figures 4A and 4B, Movies S9 and S10). Although centrosomes were reported to be stably positioned within the cell body in cultured neurons in other systems (Calderon de Anda et al., 2005, 2008), centrosomes in cultured RGCs exhibited remarkably dynamic behavior. They mainly scooted around the cell body, and could also be seen darting into neurites in some instances (Figure 4B, t = 04:00). The dynamic centrosome behavior was evident both in multipolar Stage 2 RGCs and in Stage 3/4 RGCs that had extended long axons. To test for a spatial relationship between extended axons and centrosome position, we performed centroid analysis by dividing the cell body of RGCs that had extended long axons into four quadrants relative to the base of the axon. This demonstrated that centrosome positioning is not significantly biased to any of these quadrants (Figure 4C, p = 0.9536, Chi square test, n = 33 cells).

Therefore, a simple correlation between centrosome position and neuronal polarity is not apparent in cultured RGCs, suggesting that its position is not important in this context. However, imaging of the centrosome provided a second intracellular marker that behaves differentially in the in vivo and in vitro (Stage 2) context. For this reason, we looked at centrosome behavior within RGCs in vivo, both in WT and Lamα1-deficient retinas. Blastomeres were transplanted from *ath5:GAP-RFP/Centrin-GFP* into either WT or *lamα1* morpholino-injected embryos, respectively. Consistent with previous observations (Zolessi et al., 2006), RGCs within a WT environment demonstrated static and apical centrosomal localization which persisted in maturing RGCs until the formation of the inner plexiform layer (IPL) was clearly visible, indicating that dendrites had been formed (Figure 4D, Movie S7). When developing within a Lamα1-deficient environment, the centrosomes of RGCs were initially observed in the proper position apically, at the tip of the apical process. This was seen in neuroepithelial progenitor cells and immature RGCs as *ath5:GAP-RFP* signal began to increase (Figure 4E, Movie S7). However, as RGCs matured and began to polarize within Lamα1-deficient retinas, the centrosomes of such cells often “fell” out of the apical process and moved into the cell body. Once mislocalized from the apical process, centrosomes moved dynamically within the cell body of the RGC, traveling, for example, to the basal side of the cell body and then back up apically. Therefore, Lam1 at the basal lamina in vivo is essential for the normal polarized behavior of the centrosomes, and in the absence of this extrinsic cue, polarizing RGCs behave more similarly to RGCs in vitro.

### Lam1 Is Sufficient to Orient RGC Polarization In Vivo

**Figure 5:**
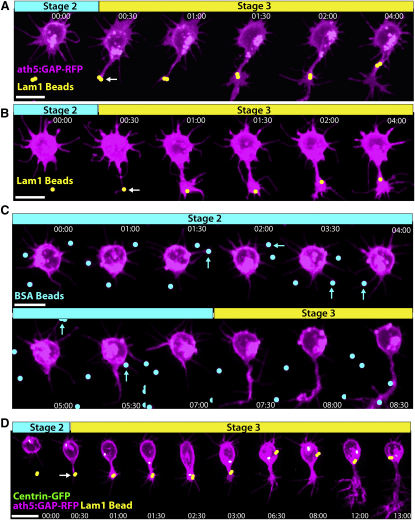
Lam1 Contact Is Sufficient to Transform a Stage 2 Neurite into an Axon In Vitro Dissociated RGCs from *ath5:GAP-RFP* embryos were plated on poly-L-lysine scattered with Lam1-coated 1 μM polystyrene beads (pseudocolored yellow) and analyzed by time-lapse microscopy. (A and B) When a Stage 2 neurite contacts a Lam1 bead (contact point marked by arrow), this quickly (within one 30 min time point) induces a dramatic transformation from a thin neurite to one with an elaborate growth cone typical of an RGC axon. (C) When presented with BSA-coated control beads (pseudocolored cyan), neurite contact (cyan arrows) does not have an observable effect. (D) Imaging of cultured *ath5:GAP-RFP/Centrin-GFP* RGCs contacting a Lam1 bead (pseudocolored yellow, arrow indicates contact point) demonstrates that along with axon induction, neurite contact causes the centrosome to orient toward the site of Lam1 contact, and induces a transient migration toward the bead. Frames are taken from Movies S8 and S9. Time is shown in hr:min; scale bars = 10 μM.

Having established that Lam1 is necessary for directed RGC axon extension, we wanted to know whether Lam1 is playing an indirect role, such as maintaining general retinal organization, or if Lam1 alone is capable of instructing this process. To address this sufficiency question, we first tested if Lam1 can direct axon extension in vitro. Polarizing RGCs plated on poly-L-lysine were presented with polystyrene beads coated with Lam1, and the influence of bead contact on polarization behavior was assessed (Figures 5A and 5B, Movie S8). When a Stage 2 neurite contacted a Lam1 bead, this induced a clear and dramatic morphological change, where the neurite transformed from a thin dynamic process to a stable process tipped with an elaborate growth cone structure typical of an axon. When long-term imaging was performed, this process consistently extended to form the axon. As a control, Stage 2 RGCs were presented with BSA-coated beads (Figure 5C, Movie S8). Contact between a BSA bead and a Stage 2 neurite had no observable effect on polarization behavior, indicating that the Laminin coating, and not the mere presence of a bead, is directing the cellular behavior. Therefore, consistent with our previous data, contact with Lam1 quickly directs the RGC polarization, and will specify a particular Stage 2 neurite to become the axon.

We next tested whether Lam1 bead contact is able to influence centrosome positioning in cultured RGCs, because Laminin is able to direct proximal centrosome localization in cultured cerebellar granule neurons (Gupta et al., 2010). We found that when a Stage 2 neurite of a cultured *ath5:GAP-RFP/Centrin-GFP* cell contacts a Lam1 bead, this quickly (within 1 hr) causes the centrosome to reorient to the Lam1 contact point (Figure 5D, Movie S9). This result is quite surprising given that RGCs extend basal axons with apical centrosomes in vivo, and that centrosome positioning does not correlate with the position of axon extension in vitro. However, we also observed that Lam1 contact can induce a subtle migration or translocation of the RGC cell body toward the bead, where the cell body appears to be tugged toward the bead. It could be that Lam1 can actually induce two separate behaviors in vitro: converting a Stage 2 neurite into an axon, and inducing cell migration/translocation, where perhaps only the latter depends on centrosome reorientation or stable centrosome positioning. In support of this, proximal centrosome localization was sometimes unstable, and the centrosome could reorient during Stage 3. This was especially obvious in cases where the bead became dislodged from its original position after neurite contact and was pulled onto the surface of the cell body. The neurite that had originally contacted the bead remained committed to form the axon, while the centrosome tracked the bead as it moved around the cell body. This indicates that Laminin-dependent axon commitment is an early event that only transiently depends on localized Laminin contact, and is separable from the persistent effect of Laminin on centrosome localization.

Although we have established that Lam1 is sufficient to direct axon commitment in vitro, we wanted to know if this was also the case in vivo. To answer this question we developed a system to implant polystyrene beads into the retina of 24 hpf zebrafish embryos using a sharp glass needle. This system allowed us to reintroduce Lam1 into a Lamα1-deficient embryo, to unambiguously identify where the ectopic Lam1 was located, and to assess its influence on polarizing RGCs. The bead implantation procedure did not have a dramatic effect on the structure of the retina, which had no noticeable structural defects, and appeared normal with a bead, or a clump of beads, suspended within it (data not shown).

**Figure 6:**
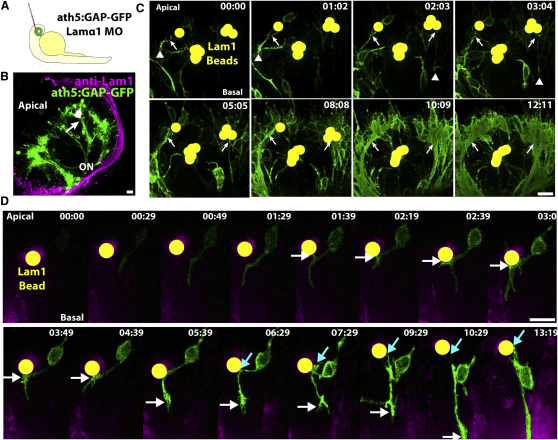
Lam1 Contact Is Sufficient to Transform a Neurite into an Axon In Vivo (A) *ath5:GAP-GFP* transgenic, *lamα1* morphant embryos were grown to ∼24 hpf, and Lam1-coated polystyrene beads were implanted into the retina of the right eye. (B) Confocal reconstruction of an embryo that was implanted with a clump of 1 μM Lam1 beads and grown until RGC axons had extended (∼3 dpf), immunostained with polyclonal anti-Lam1 antibody. This demonstrates the intimate association between the highly stained Lam1-coated beads and the extended axons. Axons hug the surface of the bead clump (arrow), causing the beads to lie within the axon fascicle. (C) Time-lapse confocal imaging during axon extension demonstrates the dramatic interaction between polarizing RGC axons and the Lam1-coated beads (pseudocolored yellow), resulting in axon extension along the surface of the beads (visible growth cones marked by arrowheads) and bead engulfment by the axon fascicle (arrows). (D) Highlighting an individual RGC by pseudocoloring it green demonstrates that after Lam1 bead contact (white arrows), the contacting neurite transforms into a process tipped with an elaborate growth cone that extends to form the axon, while the axon shaft remains associated with the bead (blue arrows). Frames are taken from Movies S10 and S12. Time is shown in hr:min; scale bars = 10 μM.

Lam1-coated beads were implanted into 24–28 hpf *lamα1* morphant embryos (Figure 6A). Embryos were grown until 3 dpf, and we imaged them by confocal microscopy to look for an interaction between the beads and RGC axons. In many cases an interaction between the beads and RGC axons was obvious, where large axon bundles were observed in contact with the beads/bead clumps. Axons hugged the surface of the beads, often causing them to lie within the axon fascicle (Figure 6B). Beads were generally positioned at the base of the RGC axon bundles, close to RGC cell bodies, consistent with the hypothesis that Lam1 is acting to direct polarization and RGC axon sprouting.

Axon growth can be directed by the physical nature of a substrate. Therefore, it is possible that the physical presence of a polystyrene bead, rather than the Lam1 coating, is able to influence RGC polarization and axon extension. To control for this possibility, we implanted BSA-coated beads into Lamα1 morphant embryos. BSA-coated beads very rarely showed an association with RGC axons. To quantify this observation, confocal stacks from retinas implanted with either Lam1 or BSA-coated beads were blinded and classified as either showing a clear and dramatic interaction with RGC axons, where many RGC axons were seen in contact with the surface of the bead (similar to those shown in Figure 6B), or not. Lam1-coated beads fell into the positive category 38.1% of the time (n = 42 beads, in 33 retinas), in contrast to only 5.5% of BSA-coated beads (n = 36 beads, in 25 retinas).

To assess this interaction in more detail, we performed time-lapse imaging experiments. After Lam1-coated bead implantation, the embryo was allowed to recover for 5–10 hr, and then imaged during the period of RGC axon extension. Most RGCs that came in contact with the surface of the Lam1 bead consistently showed a very strong interaction (Figure 6C, Movies S10 and S11). RGCs tightly associated with the beads and extended axons along their surface (70% of experiments, n = 20 beads/bead clumps, in 14 embryos). The growth cones of these axons subsequently navigated away from the bead, toward the basal surface of the retina, leaving bundles of fasciculated axons hugging the surface of the bead (arrows, Figure 6C). The RGCs generally remained associated with the beads for the length of imaging session, and the RGC layer appeared to organize itself around the Lam1 bead. In contrast to the dramatic effect of the Lam1 beads, BSA-coated beads did not show any substantial interaction with RGCs (n = 6 beads, in five embryos). Instead BSA-coated beads appeared to float aimlessly within the retina, indicating that they do not interact with any retinal cells (compare Lam1 and BSA-coated beads in Movie S11).

In some instances it was possible to track an isolated RGC as it came into contact with a Lam1-coated bead, as is shown in Figure 6D (Movie S12). This young RGC exhibited a typical morphology, with apical and basal processes. The RGC then contacted the Lam1 bead at approximately the midpoint of the basal process (yellow arrowhead). The distal portion of the basal process then retracted, and short dynamic neurites were evident at the point of Lam1 contact. The growth cone then sprouted from the contact point, and subsequently navigated away toward the retinal basal surface, demonstrating that Lam1 contact is sufficient to specify the point from which the RGC axon will emerge. The axon shaft remained associated with the bead, and was even observed to split in the example shown (blue arrowhead). This highlights the tight adherence of RGC axon to the Lam1 surface, and the critical importance of Laminin to RGC axons in vivo.

### Lam1 Contact Directs the Localized Microtubule Changes Leading to Kif5c560-YFP Accumulation

A requisite step in axon selection is the differential rearrangement of microtubules in the preaxonal neurite (Witte et al., 2008). This is likely what is visualized using the Kif5c560-YFP microtubule motor construct. Based on the coincident timing of Kif5c560-YFP signal accumulation with when the tip of the RGC basal process contacts the basal surface of the retina, as well as the lack of directed Kif5c560-YFP accumulation when RGCs lack this Lam1 cue, we hypothesized that the role of Lam1 may be to direct the localized microtubule modifications leading to the specific Kif5c560 accumulation preceding definitive axon extension. To test this, we wanted to see if we could recapitulate the normal directed behavior using our Lam1 bead assays.

**Figure 7:**
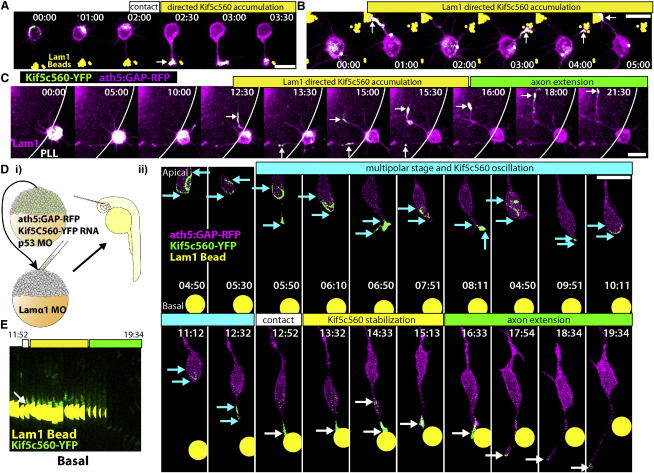
Lam1 Contact Directs Kif5c560-YFP to the Contacting Neurite In Vitro and In Vivo (A–C) RGCs were dissociated from *ath5:GAP-RFP* embryos injected with Kif5c560-YFP mRNA at the one-cell stage, plated on poly-L-lysine with scattered 1 μM Lam1-coated beads (A and B, pseudocolored yellow), or on islands of Lam1 stained with Texas red dye (C, magenta substrate). When a single neurite contacted Lam1 (A), this quickly caused the YFP signal to accumulate specifically in that neurite (arrow). When multiple neurites contacted Lam1 (B and C), this caused the YFP signal to accumulate specifically in Lam1-contacting neurites, and to oscillate preferentially between these neurites (arrows). (Di) Blastomeres were transplanted from *ath5:GAP-RFP* transgenic embryos that were injected with Kif5c560-YFP mRNA into *lamα1* morphant embryos or mosaic embryos grown to 24 hpf, and 6 μM Lam1-coated beads were implanted into the right eye. (Dii) The highlighted RGC exhibits typical Kif5c560-YFP oscillations during Stage 2 (cyan phase, marked by red arrowheads). Upon contact with the Lam1 bead (white arrowhead; bead is pseudocolored yellow), the contacting process is stabilized and does not retract, and YFP signal is concentrated at the contact point. This neurite then extends to form the axon (green phase). Note that the individual confocal z-slices were cropped to remove signal not associated with the cell. A reconstruction of the uncropped frames is shown in Movie S16. (E) Kymograph of the cell shown in (D), demonstrating the specific Kif5c560-YFP signal accumulation upon neurite contact with the Lam1-coated bead (contact marked by white arrow; bead pseudocolored yellow). Frames are taken from Movies S13, S14, S15, and S16. Time is shown in hr:min; scale bars = 10 μM.

Kif5c560-YFP-expressing RGCs were cultured in the vicinity of Lam1-coated polystyrene beads (Figure 7A, Movie S13). Consistent with our model, when a Stage 2 neurite contacted a Lam1 bead, this induced the translocation of the Kif5c560-YFP signal to the contact point, demonstrating that Laminin contact catalyzes this specific accumulation. Interestingly, when two or more neurites contacted Lam1, Kif5c560-YFP accumulated in specifically these contacting neurites, but often only in one neurite at a time, and oscillated between these (but rarely other) neurites (Figure 7B, Movie S14). RGCs were also cultured along borders of poly-L-lysine and Lam1 by plating on coverslips with islands of Lam1 within a homogenous poly-L-lysine coating. Similar to when RGCs contacted multiple Lam1 beads, an RGC polarizing along a Lam1 border demonstrated a clear bias in Kif5c560-YFP accumulations, where the signal oscillated between different Lam1-contacting neurites before stabilizing in one, which extended to form the axon (Figure 7C, Movie S15).

Having established that this was the case in vitro, we moved to the in vivo assay. Lam1 beads were implanted into mosaic embryos created by transplantation of blastomeres from *ath5:GAP-RFP*, Kif5c560-YFP RNA-injected embryos into Lamα1 morphant host embryos (Figures 7D and 7E, Movie S16). As described above, RGCs in the Lam1-deficient environment exhibit oscillatory Kif5c560-YFP accumulations. However, when one of the neurites contacted the Lam1-coated bead, Kifc560-YFP accumulated specifically at the contact point. The YFP signal accumulation was stable, with only very transient and weak signal visible within the basal process, and the Lam1 contacting process did not retract. Subsequently this neurite transformed into the axon and extended away from the bead. Therefore, contact with Lam1 caused the cessation of the Kif5c560-YFP oscillations within Stage 2 RGCs in vivo, and recapitulated the normal behavior of RGCs when they come in contact with the basal surface of the WT retina, where Lam1 contact results in specific and stable Kif5c560-YFP accumulation preceding axon extension.

## Discussion

### Lam1 Orients the Polarization of RGCs In Vivo

Imaging experiments in the vertebrate retina have demonstrated that bipolar cell polarization occurs through the directed sprouting of axons and dendrites from basal and apical processes, respectively (Morgan et al., 2006). Similarly, RGC polarization occurs through directed sprouting of axons from the most basal point of the cell. In contrast to behavior in cultured neurons, no multipolar Stage 2 behavior is seen prior to RGC axon extension in vivo. We have confirmed and extended these findings, and have shown that RGC polarization is highly directed intracellularly, where Kif5c560-YFP, a marker of axonal microtubules, is directed to accumulate at the tip of the basal process prior to axon extension. Therefore, it is clear that these retinal neurons are endowed with polarity information that is not present in cultured neurons and allows them to extend axons directly from the relevant part of the cell body. There are two potential sources of information that neurons could exploit. Neurons derive from the terminal divisions of highly polarized neuroepithelial cells, and this inherited polarity could be instructing neuronal polarization. Alternatively (or additionally) neurons are born into highly heterogeneous environments, in which multiple potential extracellular cues exist that could provide polarizing information.

Here we demonstrate that a major determinant for the orientation of RGC polarization is in fact an extracellular cue acting upon the neuron. Just prior to axon extension, the RGC contacts the basal surface of the retina. Lining the basal surface of the retina is a basal lamina, which contains Lam1. For five reasons we conclude that Lam1 contact is the major cue instructing the RGCs to extend their axons at this precise point. First, contact with the retinal basal surface correlates with the specific and stable accumulation of the axonal marker Kif5c560-YFP. Second, in a retina devoid of Lam1 at its basal surface, RGCs show ectopic polarization behaviors and progress through a Stage 2 phase before extending an axon. Third, in a Lam1-deficient retina, centrosomes were localized appropriately and apically in very young RGCs, but mislocalized and wandering centrosomes are visible within Stage 2 RGCs. This suggests that Lam1 is most crucial to direct neuronal (rather than neuroepithelial) polarization, at least with respect to this marker in these specific cells. Fourth, when cultured RGCs contact a Lam1-coated bead, they will extend their axons from the contact point. Fifth, and most importantly, when Lam1-coated beads are implanted into a Lam1 deficient retina, RGCs that contact the bead consistently extend their axon along the Lam1 surface.

This is a clear demonstration that a molecularly defined cue is necessary and sufficient to orient the polarization of a vertebrate neuron in vivo. Importantly, the role of Laminin in neuronal polarization may not be limited to RGCs, because many neurons, including hindbrain and spinal cord neurons, extend axons along basal laminae. Also, the orientation of nucMLF neuron axon extension has been reported to be disrupted in zebrafish Lamα1 mutants, and knockout of *lamγ1* in cortical neurons results in migratory and perhaps polarization defects, indicating that a Laminin-based cue may be important for directed polarization of diverse types of neurons (Chen et al., 2009; Wolman et al., 2008). Interestingly, it was recently demonstrated that rhombic lip-derived neurons in the zebrafish cerebellum exhibit apical centrosomes during polarization and show an accumulation of Kif5c560-YFP during axon extension from the basal surface of the cell, indicating that these neurons may polarize via the same mechanisms as RGCs, although this remains to be tested (Distel et al., 2010).

### Laminin Contact Directs RGC Polarization through the Stabilization of the Basal Process and Localized Changes in Microtubules

**Figure 8:**
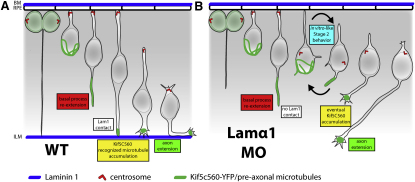
Model for Directed RGC Axon Emergence (A) In a WT retina, the newly born RGC re-extends a basal process toward the basal surface of the retina. The process contacts Lam1 within the retinal basal lamina at the ILM (blue). This stabilizes the basal process, causes Kif5c560-YFP-recognized microtubules (green) to accumulate specifically at the contact point, and commits this neurite to form the axon. (B) In a Lam1-deficient retina, the re-extending basal process does not contact the Lam1 cue, causing it to retract, and causing the cell to enter an ectopic Stage 2 phase typical of neurons polarizing in vitro. During this phase transient neurites are extended, Kif5c560-YFP signal oscillates within the cell, and the centrosome becomes mislocalized and dynamic. Driven by the intrinsic polarization program, and perhaps by other intrinsic/extrinsic cues, Kif5c560-YFP signal eventually stabilizes in a single neurite that matures and extends to form the axon.

Although they are clearly demonstrating disrupted polarization behaviors, RGCs within a Lam1-deficient retina rarely invert, and Kif5c560-YFP only ever localizes to basally directed neurites within Stage 2 RGCs. Therefore, while these cells do exhibit a polarization behavior more similar to that of cultured neurons, complete intracellular (or morphological) inversions rarely occur. Thus, other extracellular cues that prevent apical Kif5c560-YFP accumulations and RGC inversions may be present in the retina (Bauch et al., 1998; Zolessi et al., 2006). Alternatively, there may be an intrinsic polarity to the RGCs, which is independent of Lam1 and acts to prevent RGC inversions. At present we are unable to differentiate between these two possibilities. However, since Kif5c560-YFP accumulates basally in neuroepithelial cells, this seems to lend support to the latter possibility. We propose that there are likely to be multiple factors that are directing the polarization of RGCs, acting independently of Laminin to prime RGCs to polarize toward the basal surface. Lam1 then acts as the final cue, defining the precise point where the axon will emerge, and committing the axon to sprout at the contact point (Figure 8). Laminin contact occurs so soon after RGC birth that it directs the maturation into Stage 3 before the cell has a chance to enter Stage 2. Our in vitro and in vivo Lam1 bead assays indicate that this occurs through the capture and stabilization of the contacting process (normally the re-extending basal process), and the direction or reinforcement of the localized changes in microtubules, resulting in Kif5c560 accumulation and axon extension. In the absence of Lam1, this rapid transition to Stage 3 does not occur, and RGCs revert to Stage 2.

Exactly how Laminin contact influences microtubules in this context is not clear. Perhaps telling is the observation that when multiple neurites of cultured RGCs are contacting Lam1, Kif5c560-YFP oscillates only between these neurites. This demonstrates that whether or not a neurite contacts Laminin somehow differentially influences the microtubules of that neurite, so that its capacity to accumulate Kif5c560-YFP is altered. This could occur through the formation of a more ordered array of microtubule plus ends aligned at the tip of the neurite, resulting in more localized accumulations of the plus-end-directed Kinesin 1 motor. Alternatively, the specific recruitment of MAPs or tubulin-modifying enzymes to the Lam1 contact point could direct biochemical changes thought to direct Kif5c560 accumulation in mature axons (Hammond et al., 2010; Konishi and Setou, 2009).

Laminin contact can also direct axon extension in cultured hippocampal neurons, and perhaps cerebellar granule neurons (Esch et al., 1999; Gupta et al., 2010; Ménager et al., 2004). This Laminin signal is generally received through Integrin receptors on the cell surface. We designed and tested morpholinos against the candidate Integrin Laminin receptors: Itgβ1a, β1b, and α-6. These morphants showed phenotypes consistent with β1/α6 Integrins being the relevant receptor on the RGCs, with disorganized axon bundles within the retina, and Stage 2 RGCs during polarization (data not shown). However, because Integrins are required for many developmental events, these morphants exhibited a dramatic amount of retinal disorganization, and we were unable to specifically attribute these phenotypes to a cell-autonomous lack of Laminin responsiveness. However, previous studies have demonstrated that laminin-dependent neurite extension occurs through Integrin receptors (Gupton and Gertler, 2010), and expression of dominant-negative β1 Integrin constructs prevents axon extension in *Xenopus* RGCs (Lilienbaum et al., 1995), providing a strong indication that this is the relevant receptor for RGCs to respond to Lam1. In the future it will be important to determine precisely how the localized cytoskeletal rearrangements leading to Kif5c560 accumulation and axon commitment are directed by Lam1 contact, because an analogous mechanism is likely to have occurred for every polarized neuron in the brain.

### A Localized Lam1 Cue in the Retina Causes the Discrepancy between RGC Polarization Behavior In Vivo and In Vitro

Determining how neurons polarize in vivo is challenging due to the requirement for precise genetic manipulation of generally pleiotropic genes, and the detailed analysis of intricate cell behaviors within their often-prohibitive location deep within the developing embryonic brain. For these reasons, most of the research on neuronal polarization has been done on neurons polarizing in culture. Unlike RGCs, cortical neurons polarizing in vivo do appear to progress through a multipolar stage (Noctor et al., 2004), which has been likened to the multipolar Stage 2 of cultured hippocampal neurons (Barnes and Polleux, 2009; Calderon de Anda et al., 2010). One interpretation of this contradiction could be that the mechanism of RGC polarization is intrinsically different than that of mammalian cortical neurons. However, cultured RGCs exhibit the classical staged series of behaviors typical of cultured hippocampal neurons (Zolessi et al., 2006). We have further demonstrated that Kif5c560-YFP exhibits the transient oscillations in different areas of the cell body and Stage 2 neurites before stably accumulating in the axon just prior to extension, as was previously shown in cultured hippocampal neurons (Jacobson et al., 2006). Therefore, these two types of neurons are actually behaving identically in culture, at least at the fundamental level of the microtubule cytoskeleton, indicating that they are actually quite similar intrinsically. The major difference between these two systems appears to be one of environmental context, rather than intrinsic cellular differences, because removal of the endogenous cue, Laminin, causes polarizing RGCs within morphant retinas to switch their behavior to that of cultured neurons. In vitro polarization appears to represent the behavior of cells without their relevant cues, and there may be striking differences between this behavior and what actually occurs within the developing brain. Therefore, despite the experimental challenges, determining the molecular mechanisms governing the polarization of diverse neuron cell types in vivo will be critical to understanding how this process is actually regulated within the embryonic brain.

## Experimental Procedures

### Animals

Zebrafish were maintained and bred at 26.5°C, and embryos were raised at 28°C–32°C and staged based on hpf. Embryos were treated with 0.003% phenylthiourea (Sigma) from 10 hpf to prevent pigmentation. All animal work was approved by Local Ethical Review Committee at the University of Cambridge and performed according to the protocols of project license PPL 80/2198.

### Transgenic Lines and Constructs

Transgenic lines *Tg(atoh7:gap43-EGFP)_cu1_* and *Tg(atoh7:gap43-mRFP1)_cu2_* have been previously described (Zolessi et al., 2006), and are abbreviated here as *ath5:GAP-GFP* and *ath5:GAP-RFP*. The *Tg(Centrin-GFP)* line was created using the pCJW266 plasmid, where the β-actin promoter drives expression of zebrafish centrin fused to GFP, all flanked by ISce-1 sites (Zolessi et al., 2006). This construct was injected along with ISce-1 enzyme into one-cell stage *ath5:GAP-RFP* embryos to obtain a double transgenic line with ubiquitous Centrin-GFP expression. The coding sequence of Kif5c560-YFP was subcloned into the BamH1 and EcoR1 sites of PCS2+ by PCR amplification of the coding region from pBa-Kif5c560-YFP (Jacobson et al., 2006), using the following primer pairs: 5′-GGGGGATCCATGGCAGATCCAGCCGAATG-3′ (frw) and 5′-CCCGAATTCTTAGACGGTCCGCTTGTACAGCTC-3′ (rev). RNA was created by linearizing with Not1 enzyme and synthesizing capped RNA from the Sp6 promoter using mMessage mMachine SP6 Kit (Ambion).

### Embryo Manipulations

RNA and morpholinos were injected into the yolk of one- to two-cell stage embryos. One-half to one nanogram of Lamα1 morpholino (5′-TCATCCTCATCTCCATCATCGCTCA-3′, Gene Tools) was injected. For blastomere transplantations, high- to oblong-stage embryos were dechorionated by pronase digestion (Sigma) and placed in agarose molds, and between 5 and 30 blastomeres were transferred between embryos using a glass capillary connected to a 2 ml syringe. In most transplantation experiments, the *p53* morpholino (5′-GCGCCATTGCTTTGCAAGAATTG-3′, Gene Tools) was injected into donor embryos to prevent apoptosis of donor cells and increase the success rate of transplanted cell survival. This was especially important for transplantation from Kif5c560-YFP RNA-injected donors because this construct exhibited a mild degree of cellular toxicity. H2B-RFP/GFP RNA was generally injected as a lineage tracer to screen embryos for successful transplantations.

For bead implantation experiments, 6 μM fluoresbrite polychromatic red dyed beads, or 1 μM polystyrene beads (Polysciences), were coated by incubation with murine Lam1 (L2020, Sigma) or BSA (A2153, Sigma) for 1 hr at room temperature, and washed three times for 5 min in PBS. Efficient Lam1 coating was obtained as the Lam1-coated beads clumped together and formed aggregates, which was not seen for BSA or uncoated beads, and confirmed by strong Lam1 staining by immunofluorescence. Bead implantations were performed by mounting 24 hpf embryos in 2%–4% methylcellulose (Sigma), containing 0.4 mg/ml MS222 (Sigma) as anesthetic. Beads were suspended in the methylcellulose, sucked into a sharp glass capillary connected to a mineral-oil filled Hamilton syringe, and injected into the retina of the embryo. Embryos were then transferred to clean Petri dishes containing embryo medium and penicillin/streptomycin/fungicide to recover. The polychromatic red dye showed extremely bright fluorescence, and the signal bleedthrough into the green channel was strong enough for bead visualization in most experiments. When imaging in red channel was also necessary, beads were photobleached by being placed on the windowsill for 2–4 weeks.

Dissociated retinal cell culture was performed as previously described (Zolessi et al., 2006). For the creation of a substrate with Laminin islands, coverslips were coated overnight with poly-L-lysine (Sigma, 10 μg/ml), and then sprayed with an atomizer creating a fine mist of Lam1 (Sigma, 20 μg/ml) mixed with Texas-red-conjugated Dextran (D-1863, Invitrogen) in order to stain the Laminin deposits.

### Confocal Imaging and Immunostaining

Imaging of live and fixed embryos was performed as described previously (Poggi et al., 2005), using a Perkin Elmer Spinning Disk UltraVIEW ERS, Olympus IX81 Inverted microscope and 60× (1.2 NA) water immersion objective, and a motorized XY stage (H117, Prior) to allow for simultaneous imaging of multiple embryos. A confocal laser scanning microscope (Leica) and 63× (1.2 NA) water immersion objective (Leica) were also used for experiments shown in 3A–3C and 6B. Optical sections at 0.75–1 μm separation were taken to cover the majority of the retina (between 40 and 100 μm) at the relevant time intervals. Whole-mount immunostaining was performed using standard methods, using rabbit polyclonal anti-Lam1 (L9393, Sigma, 1:100) and anti-rabbit Alexa-594 (Invitrogen, 1:1000).

### Data Analysis

Confocal data was analyzed using Volocity (Improvision). Deconvolution was generally performed on data acquired by spinning disk confocal microscopy using the Iterative Restoration tool at 25 iterations and 99.99% confidence levels. Unless otherwise stated, the confocal z-slices were cropped to a rectangular region containing the cells of interest in XYZ and reconstructed using 3D Opacity. Brightness, contrast, and gamma were adjusted for maximal visibility of cellular morphology and fluorescent signal using Volocity, Photoshop (Adobe), and ImageJ (NIH), and the RFP channel was converted to magenta using the channels tool in ImageJ. Pseudocoloring and cell tracing was done in Photoshop, and the outline of the cell was determined by comparing it to the original confocal z-slices. For data presented in Figures 3Eii and 7Dii, each confocal z-slice were cropped tightly to the cell surface in all three spatial dimensions using Photoshop, carefully removing signal from the images not associated with that cell. The stack was then flattened into a maximum z-projection using ImageJ. For quantifications presented in Figure 2, lengths were measured within the original confocal z-slices using the line tool in Volocity. Statistical tests were performed using InStat (GraphPad).

## Supplemental Information

Movie S1. Kif5c560-YFP Accumulations Oscillate during Stage 2 and Stabilize in the Extending Axons of RGCs Polarizing In Vitro (A and B) Retinal ganglion cells (RGCs) were dissociated from *ath5:GAP-RFP* (magenta channel) embryos injected with Kif5c560-YFP mRNA (green channel) at the one-cell stage and imaged during polarization in vitro. Shortly after plating, RGCs exhibit Kif5c560-YFP oscillations in different areas of the cell body and individual neurites. YFP signal eventually stabilizes in a single neurite that extends to form the axon. Time is shown in hr:min:s:ms.

Movie S2. Kif5c560-YFP Accumulation Is Highly Directed In Vivo, and Accumulates at the Tip of the Basal Process Prior to Axon Extension Time-lapse confocal imaging on mosaic embryos with RGCs from *ath5:GAP-RFP* (magenta channel) and Kif5c560-YFP (green channel) within WT embryos. Prior to specific accumulation in the extending axonal growth cones (white arrowheads), Kif5c560-YFP signal initially spans a large portion of the basal process (magenta arrowheads), but upon contact with the basal surface, the YFP signal accumulates specifically at the tip of the basal process (white arrowheads), and finally in the extending axon. Time is shown in hr:min:s:ms.

Movie S3. Lamα1 Is Required for Highly Directed RGC Polarization Time-lapse confocal imaging of mosaic embryos with WT *ath5:GAP-GFP*-labeled RGCs within *lamα1* morphant embryos. In this Lam1-deficient environment, RGCs can be seen progressing through a transient multipolar phase (marked by [^∗^]; cells 1, 3, and 4) and project axons at orientations other than directly basal (marked by arrowheads). Time is shown in hr:min:s:ms.

Movie S4. Lamα1 Is Dispensable within RGCs for Highly Directed Polarization (A and B) Time-lapse confocal imaging of mosaic embryos with *lamα1* morphant *ath5:GAP-GFP*-labeled RGCs within a WT environment. In this context morphant RGCs polarize normally and axons (marked by arrowheads) project directly from the most basal point of the cell. Time is shown in hr:min:s:ms.

Movie S5. In a Lamα1-Deficient Environment, Polarizing RGCs Exhibit Oscillations in Kif5c560-YFP Signal Accumulation Typical of Stage 2 Neurons Polarizing In Vitro Time-lapse confocal microscopy of mosaic embryos with WT *ath5:GAP-RFP*-labeled (magenta channel), Kif5c560-YFP-expressing (green channel) RGCs within a *lamα1* morphant environment. In this context, Kif5c560-YFP signal accumulation (marked by magenta arrowheads in RGC outlined in white) oscillates between the cell body and transient neurites before stably accumulating in a single neurite (marked by white arrowhead) that extends to form the axon. Note that the highlighted cell resides within a clone of multiple Kif5c560-YFP-expressing, transplanted cells, and arrowheads mark the relevant YFP signal that is within the highlighted RGC. Time is shown in hr:min:s:m*s.*

Movie S6. Centrosomes Are Dynamic in RGCs Polarizing In Vitro (A and B) RGCs from *ath5:GAP-RFP/Centrin-GFP* (magenta channel/green channel, respectively) transgenic embryos were dissociated and imaged during polarization in vitro, demonstrating that centrosomes are neither static nor localized to any quadrant within the cell. Instead, centrosomes move dynamically within the neuron during Stage 2, and after the axon has extended. Time is shown in hr:min:s:ms.

Movie S7. Centrosomes Are Static and Apically Localized in RGCs Polarizing in WT Retinas, but Mislocalized and Dynamic in RGCs Polarizing *lamα1* Morphant Retinas (A) Time-lapse confocal imaging of mosaic embryos with WT *ath5:GAP-RFP/Centrin-GFP-* (magenta channel/green channel, respectively) labeled RGCs within a WT environment. Centrosomes (arrowheads) remain apically positioned in RGCs throughout axon extension and up until dendrite formation and IPL stratification. Time is shown in hr:min:s:ms. (B) Time-lapse confocal imaging of mosaic embryos with WT *ath5:GAP-RFP/Centrin-GFP*-labeled RGCs within a *lamα1* morphant environment. The centrosome (arrowheads) becomes mislocalized during RGC polarization, and moves dynamically within the cell body. Time is shown in hr:min:s:ms.

Movie S8. Contact with Lam1-Coated Beads Rapidly Transforms a Neurite into a Growth Cone-Tipped Axon In Vitro (A and B) Dissociated *ath5:GAP-RFP*-expressing RGCs were plated on poly-L-lysine scattered with Lam1-coated 1 μM polystyrene beads (visible by autofluorescence, white circles at t = 0:00). Contact with a Lam1-coated bead induces the rapid transformation of the process into a growth cone-tipped neurite typical of an RGC axon. (C) In contrast, contact with BSA-coated control beads (green circles at t = 00:00) has no observable influence on the contacting neurite. Time is shown in hr:min:s:ms.

Movie S9. Neurite Contact with Lam1 Causes Centrosome Reorientation and Somal Translocation toward Lam1 In Vitro, as well as Axon Induction Dissociated *ath5:GAP-RFP/Centrin-GFP-* (magenta channel/green channel, respectively) expressing RGCs were plated on poly-L-lysine scattered with Lam1-coated 1 μM polystyrene beads (visible by autofluorescence). The Stage 2 neurite contacts a clump of two beads (t = 1:30, circle) and quickly transforms into an axon-like neurite. Contact also causes the centrosome to reorient to the contact point, and the soma to translocate toward Lam1. The Lam1 bead clump then becomes dislodged from the PLL substrate and is pulled toward the cell body, where it moves dynamically around the surface of the cell. The centrosome remains approximately adjacent to the traveling beads, while the neurite that had originally contacted Lam1 extends to form the axon. Time is shown in hr:min:s:ms.

Movie S10. Lam1 Is Sufficient to Orient RGC Axon Extension In Vivo (Part 1) *ath5:GAP-GFP* transgenic embryos were injected with *lamα1* morpholino to remove endogenous Lam1. At ∼24 hpf 6 μM polystyrene beads coated with Lam1 were implanted into the retina. Beginning at ∼35 hpf these retinas were imaged by time-lapse confocal microscopy. The Lam1 bead clumps (visible as round circles in the green channel, circled in white at t = 0:00 and labeled 1, 2, and 3) have a dramatic organizational effect on the surrounding RGCs. RGCs become tightly associated with the beads, and axons project along the surface of the bead, eventually causing the bead to be engulfed by the axon fascicle. Time is shown in hr:min:s:ms.

Movie S11. Lam1 Is Sufficient to Orient RGC Axon Extension In Vivo (Part 2) Comparing sibling embryos implanted with either BSA-coated control beads (left panel) or Lam1-coated beads (right panel), and imaging them during the same time-lapse session, demonstrates that while Lam1-coated beads interact dramatically with polarizing RGCs, BSA-coated beads do not show any observable interaction, and instead float within the retina. Time is shown in hr:min:s:ms.

Movie S12. Lam1 Contact Transforms a Neurite into an Axon In Vivo *ath5:GAP-GFP* transgenic *lamα1* morphant embryos were implanted with Lam1-coated 6 μM polystyrene beads at ∼24 hpf and imaged by time-lapse confocal microscopy beginning at ∼35 hpf. An individual RGC was pseudocolored in green (left panel; right panel shows original images). After Lam1 contact (yellow arrowhead), the contacting neurite transforms into a process tipped with an elaborate growth cone that extends to form the axon, while the axon shaft remains associated with the bead (blue arrowhead). Time is shown in hr:min:s:ms.

Movie S13. Contact with Lam1 Directs Specific Kif5c560-YFP Accumulation at the Tip of a Neurite In Vitro (Part 1) Retinas from *ath5:GAP-RFP* (magenta) embryos injected with Kif5c560-YFP RNA (green) at the one-cell stage were dissociated and plated on poly-L-lysine with scattered Lam1-coated 1 uM polystyrene beads (visible by autofluorescence, circled in white at t = 0:00). Contact of a neurite with a clump of Lam1 beads induces the translocation of YFP signal to the contact point. Time is shown in hr:min:s:ms.

Movie S14. Contact with Lam1 Directs Specific Kif5c560-YFP Accumulation at the Tip of a Neurite In Vitro (Part 2) When an RGC happened to contact multiple clumps of Lam1 beads, Kif5c560-YFP oscillated specifically between these Lam1-contacting neurites. Time is shown in hr:min:s:ms.

Movie S15. Contact with Lam1 Directs Specific Kif5c560-YFP Accumulation at the Tip of a Neurite In Vitro (Part 3) As an alternative strategy, RGCs were plated on coverslips with islands of Lam1 (labeled with Texas red Dextran, magenta substrate) within a homogenous coating of poly-L-lysine, resulting in sharp Lam1/PLL borders. RGCs polarizing along a border had multiple neurites contacting Lam1, and Kif5c560-YFP signal oscillated almost exclusively between Lam1-contacting neurites before one of these neurites extended to form the axon. Time is shown in hr:min:s:ms.

Movie S16. Lam1 Contact Directs Stable and Specific Kif5c560-YFP Accumulation in the Contacting Neurite Followed by Axon Extension In Vivo Mosaic embryos with WT *ath5:GAP-RFP*-labeled (magenta channel), Kif5c560-YFP-expressing (green channel) RGCs within a *lamα1* morphant environment were implanted with Lam1-coated beads at ∼24 hpf and imaged by time-lapse confocal microscopy beginning at ∼35 hpf. As *ath5:GAP:RFP* signal begins to increase, this cell exhibits Stage 2 behavior with oscillating Kif5c560-YFP accumulations (double arrowheads). This cell then extends a neurite that comes in contact with the Lam1-coated bead (single arrowhead, bead marked “B” at t = 0:00), causing the stabilization of this process and specific and stable Kif5c560-YFP accumulation. This process then extends to form the axon. Time is shown in hr:min:s:ms.
