# Supplementary figures and images for: The Oriented Emergence of Axons from Retinal Ganglion Cells Is Directed by Laminin Contact In Vivo
Source: Neuron. 2011 Apr 28;70(2):266–80. doi: 10.1016/j.neuron.2011.03.013 (PMC3087191; doi:10.1016/j.neuron.2011.03.013)

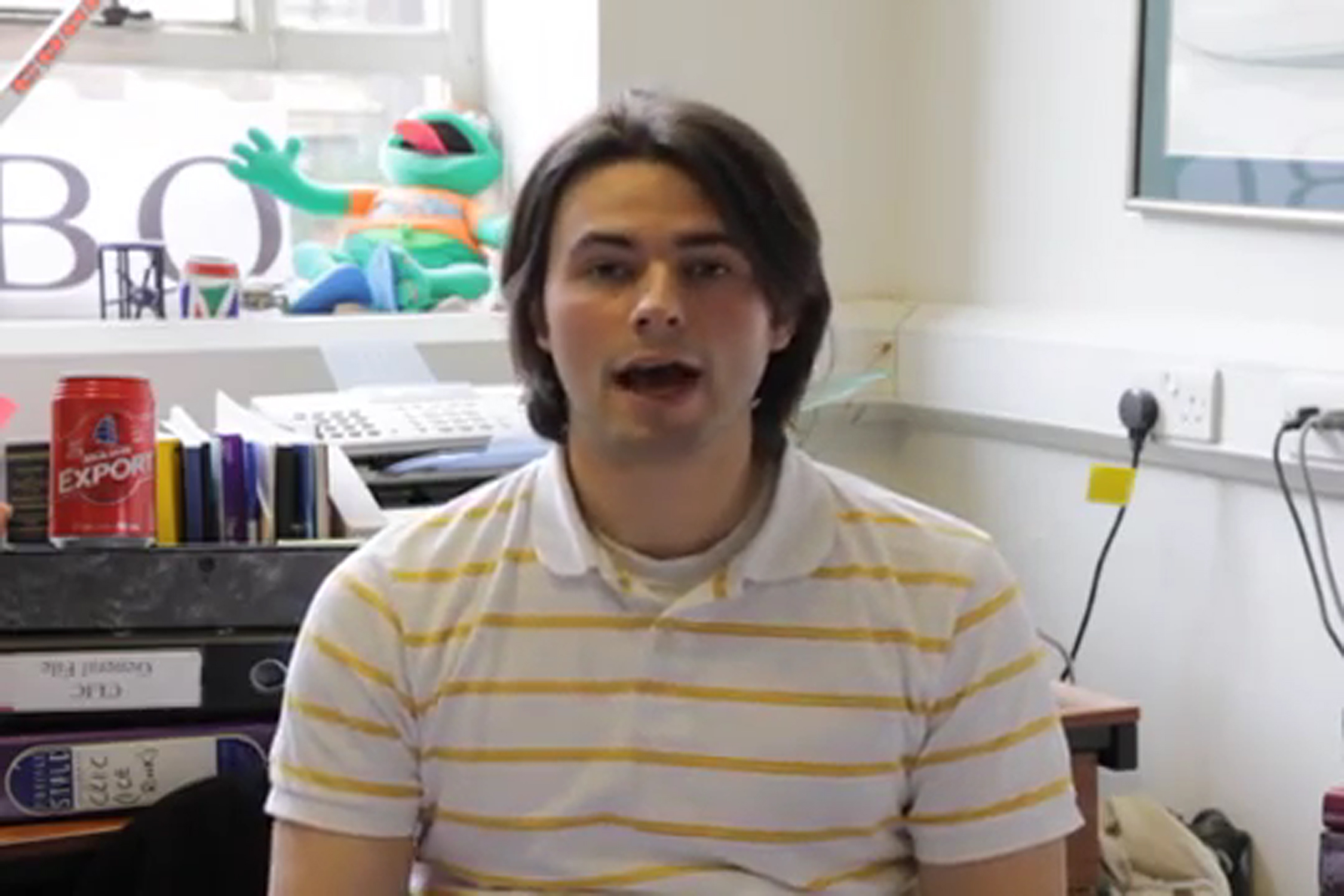

Supplement: Supplementary file 1 [file mmc17.jpg]

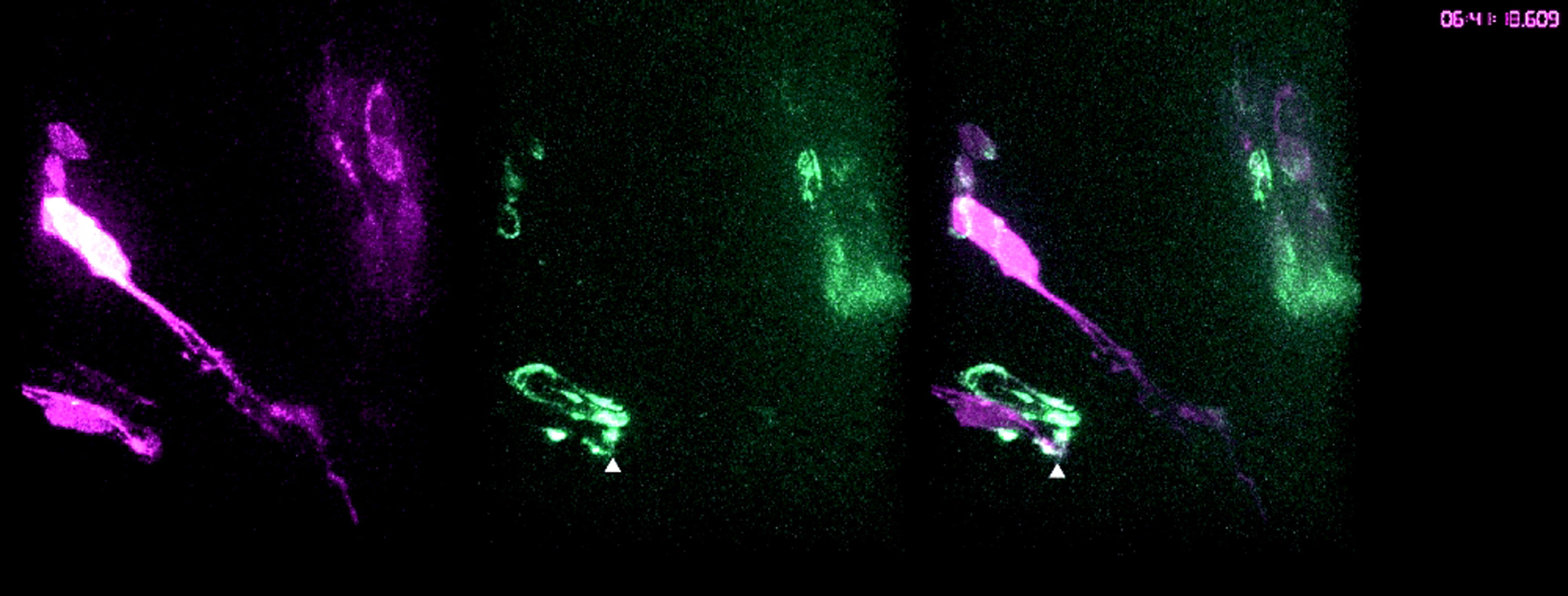

Supplement: Movie S2. Kif5c560-YFP Accumulation Is Highly Directed In Vivo, and Accumulates at the Tip of the Basal Process Prior to Axon Extension — Time-lapse confocal imaging on mosaic embryos with RGCs from ath5:GAP-RFP (magenta channel) and Kif5c560-YFP (green channel) within WT embryos. Prior to specific accumulation in the extending axonal growth cones (white arrowheads), Kif5c560-YFP signal initially spans a large portion of the basal process (magenta arrowheads), but upon contact with the basal surface, the YFP signal accumulates specifically at the tip of the basal process (white arrowheads), and finally in the extending axon. Time is shown in hr:min:s:ms. [file mmc2.jpg]

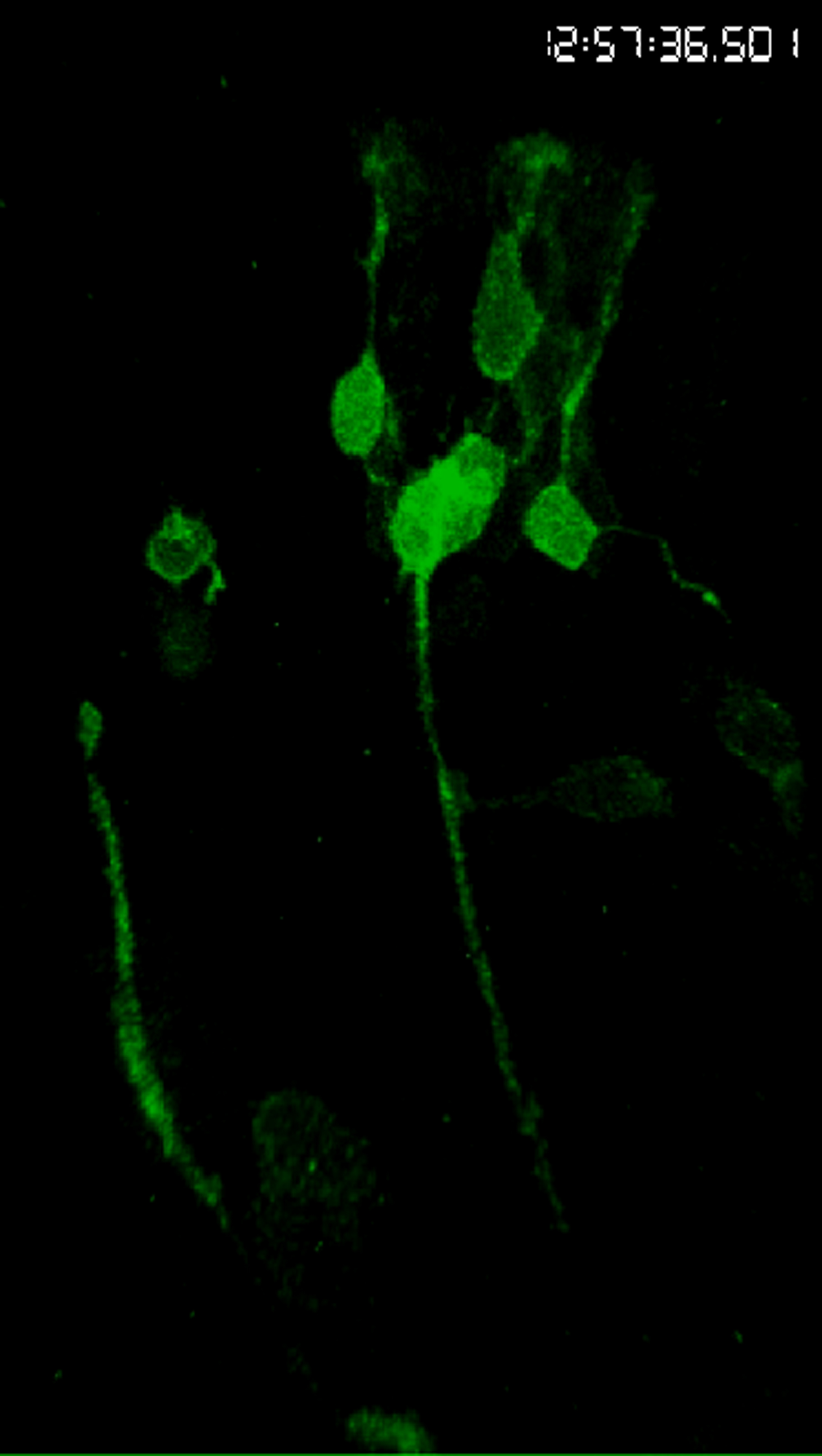

Supplement: Movie S3. Lamα1 Is Required for Highly Directed RGC Polarization — Time-lapse confocal imaging of mosaic embryos with WT ath5:GAP-GFP-labeled RGCs within lamα1 morphant embryos. In this Lam1-deficient environment, RGCs can be seen progressing through a transient multipolar phase (marked by [∗]; cells 1, 3, and 4) and project axons at orientations other than directly basal (marked by arrowheads). Time is shown in hr:min:s:ms. [file mmc3.jpg]

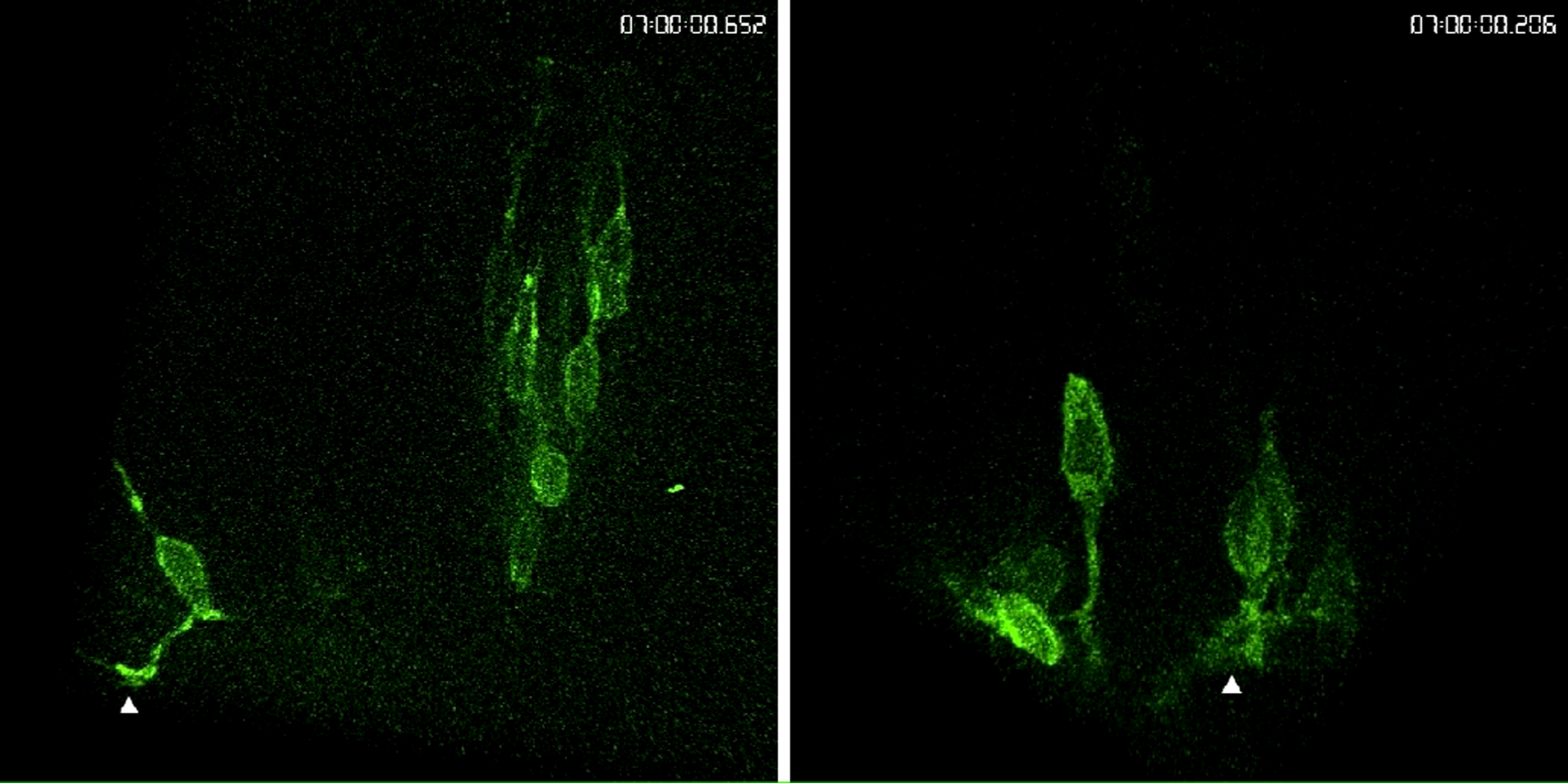

Supplement: Movie S4. Lamα1 Is Dispensable within RGCs for Highly Directed Polarization — (A and B) Time-lapse confocal imaging of mosaic embryos with lamα1 morphant ath5:GAP-GFP-labeled RGCs within a WT environment. In this context morphant RGCs polarize normally and axons (marked by arrowheads) project directly from the most basal point of the cell. Time is shown in hr:min:s:ms. [file mmc4.jpg]

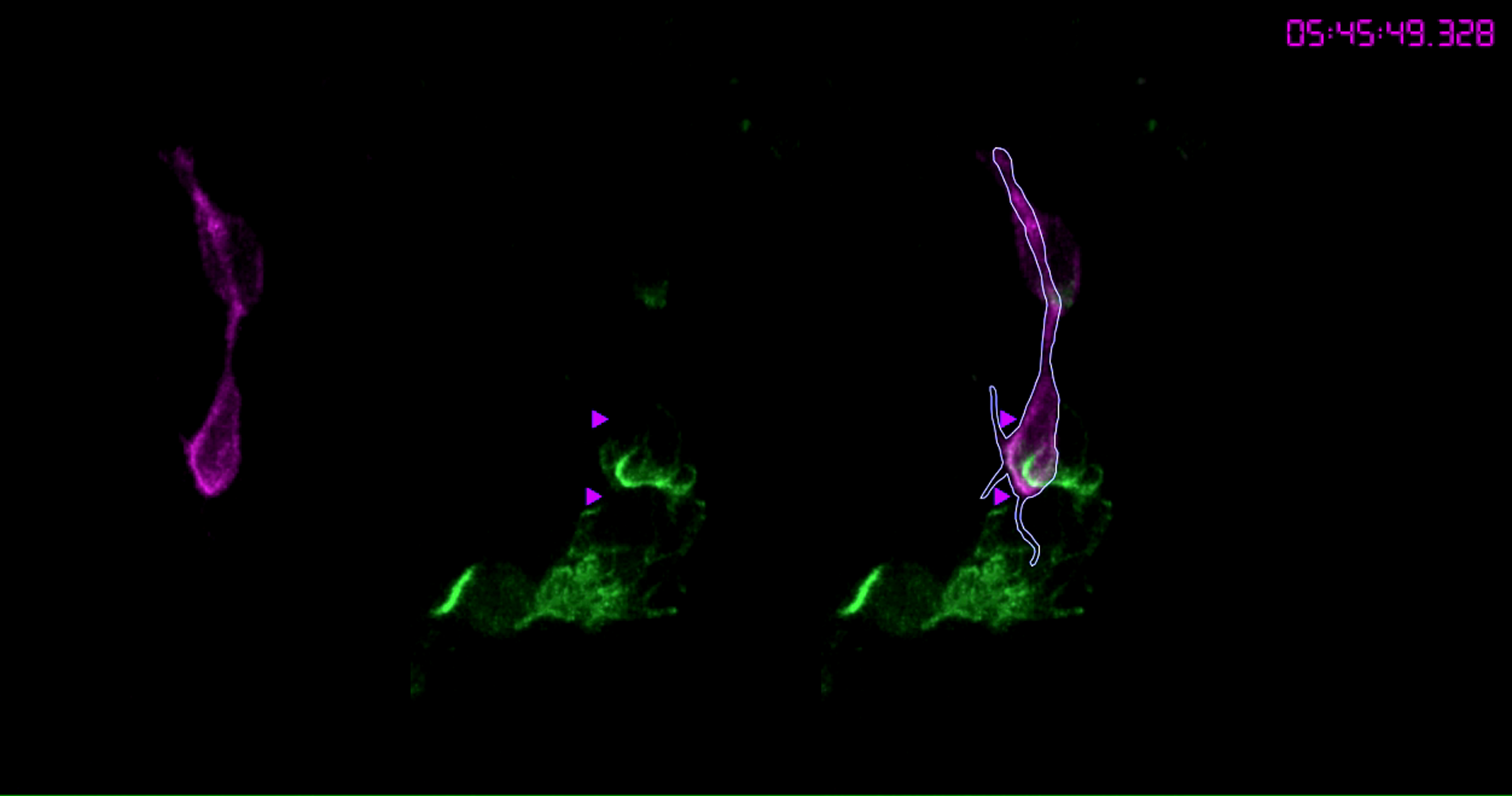

Supplement: Movie S5. In a Lamα1-Deficient Environment, Polarizing RGCs Exhibit Oscillations in Kif5c560-YFP Signal Accumulation Typical of Stage 2 Neurons Polarizing In Vitro — Time-lapse confocal microscopy of mosaic embryos with WT ath5:GAP-RFP-labeled (magenta channel), Kif5c560-YFP-expressing (green channel) RGCs within a lamα1 morphant environment. In this context, Kif5c560-YFP signal accumulation (marked by magenta arrowheads in RGC outlined in white) oscillates between the cell body and transient neurites before stably accumulating in a single neurite (marked by white arrowhead) that extends to form the axon. Note that the highlighted cell resides within a clone of multiple Kif5c560-YFP-expressing, transplanted cells, and arrowheads mark the relevant YFP signal that is within the highlighted RGC. Time is shown in hr:min:s:ms. [file mmc5.jpg]

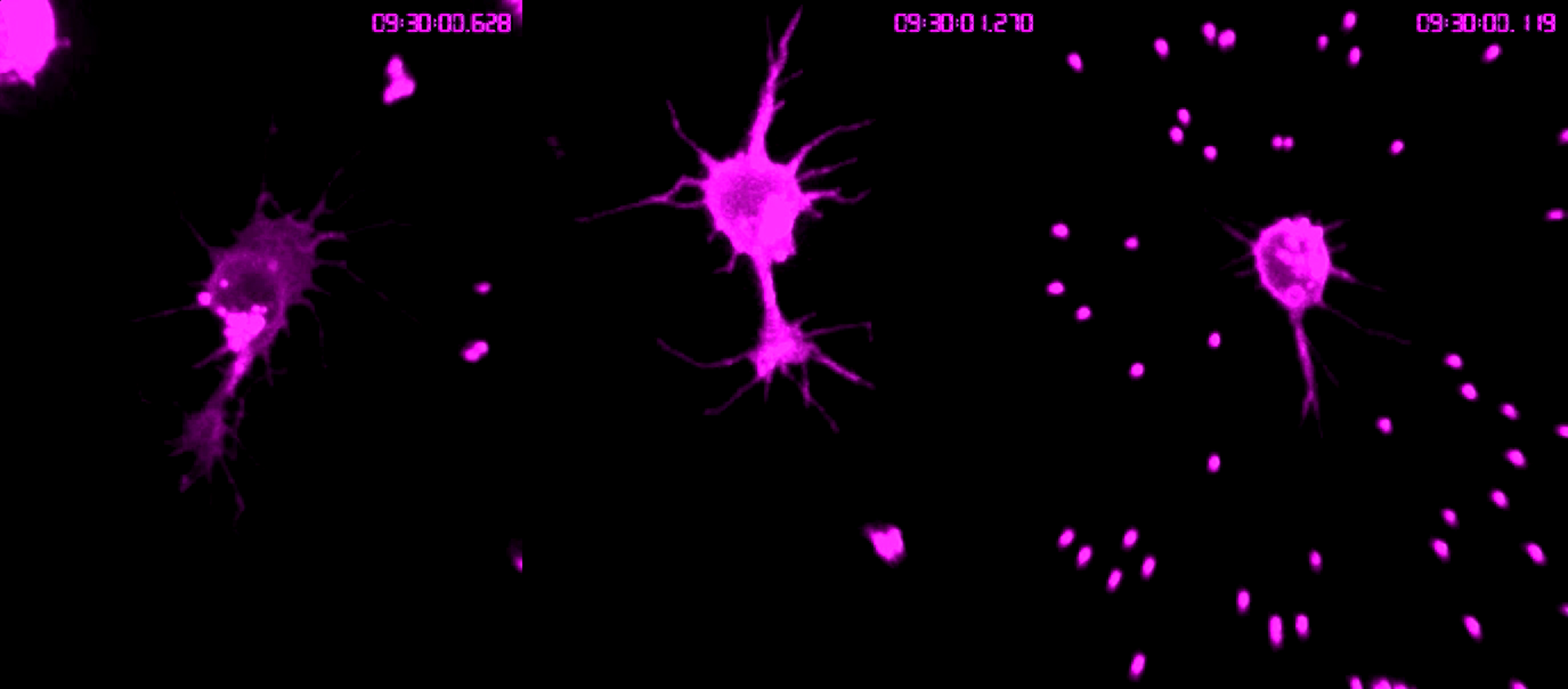

Supplement: Movie S8. Contact with Lam1-Coated Beads Rapidly Transforms a Neurite into a Growth Cone-Tipped Axon In Vitro — (A and B) Dissociated ath5:GAP-RFP-expressing RGCs were plated on poly-L-lysine scattered with Lam1-coated 1 μM polystyrene beads (visible by autofluorescence, white circles at t = 0:00). Contact with a Lam1-coated bead induces the rapid transformation of the process into a growth cone-tipped neurite typical of an RGC axon. (C) In contrast, contact with BSA-coated control beads (green circles at t = 00:00) has no observable influence on the contacting neurite. Time is shown in hr:min:s:ms. [file mmc8.jpg]

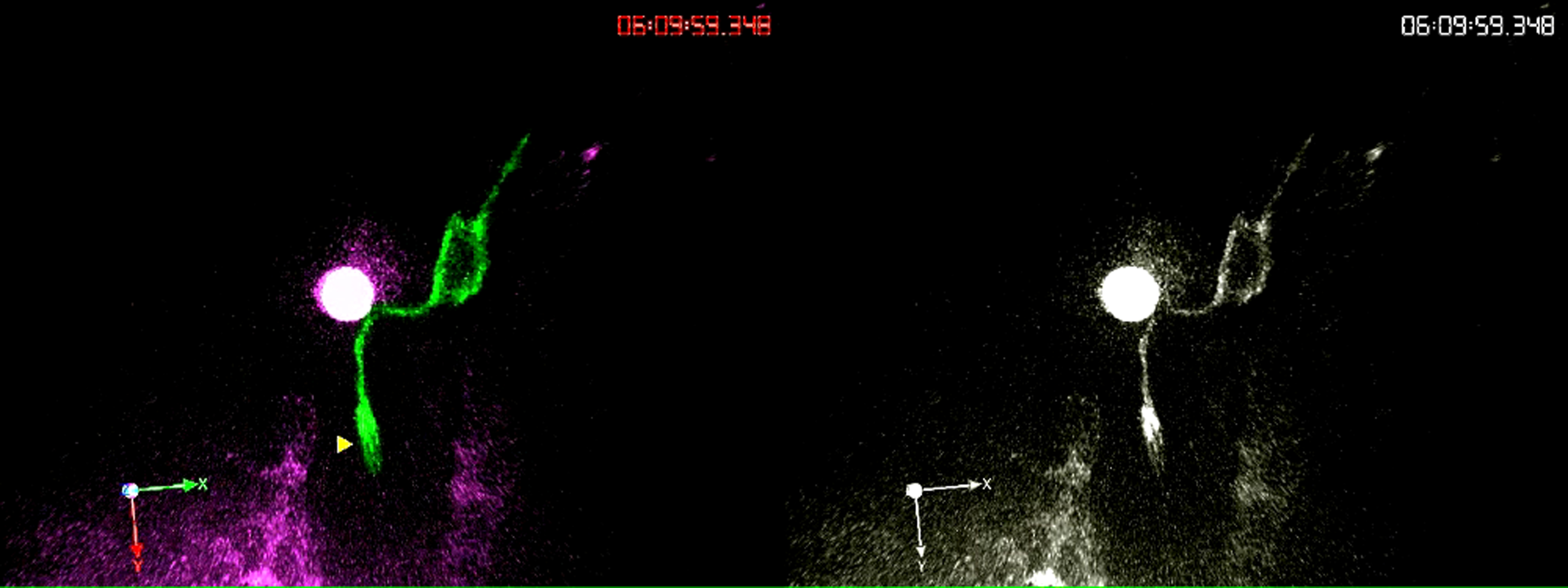

Supplement: Movie S12. Lam1 Contact Transforms a Neurite into an Axon In Vivo — ath5:GAP-GFP transgenic lamα1 morphant embryos were implanted with Lam1-coated 6 μM polystyrene beads at ∼24 hpf and imaged by time-lapse confocal microscopy beginning at ∼35 hpf. An individual RGC was pseudocolored in green (left panel; right panel shows original images). After Lam1 contact (yellow arrowhead), the contacting neurite transforms into a process tipped with an elaborate growth cone that extends to form the axon, while the axon shaft remains associated with the bead (blue arrowhead). Time is shown in hr:min:s:ms. [file mmc12.jpg]

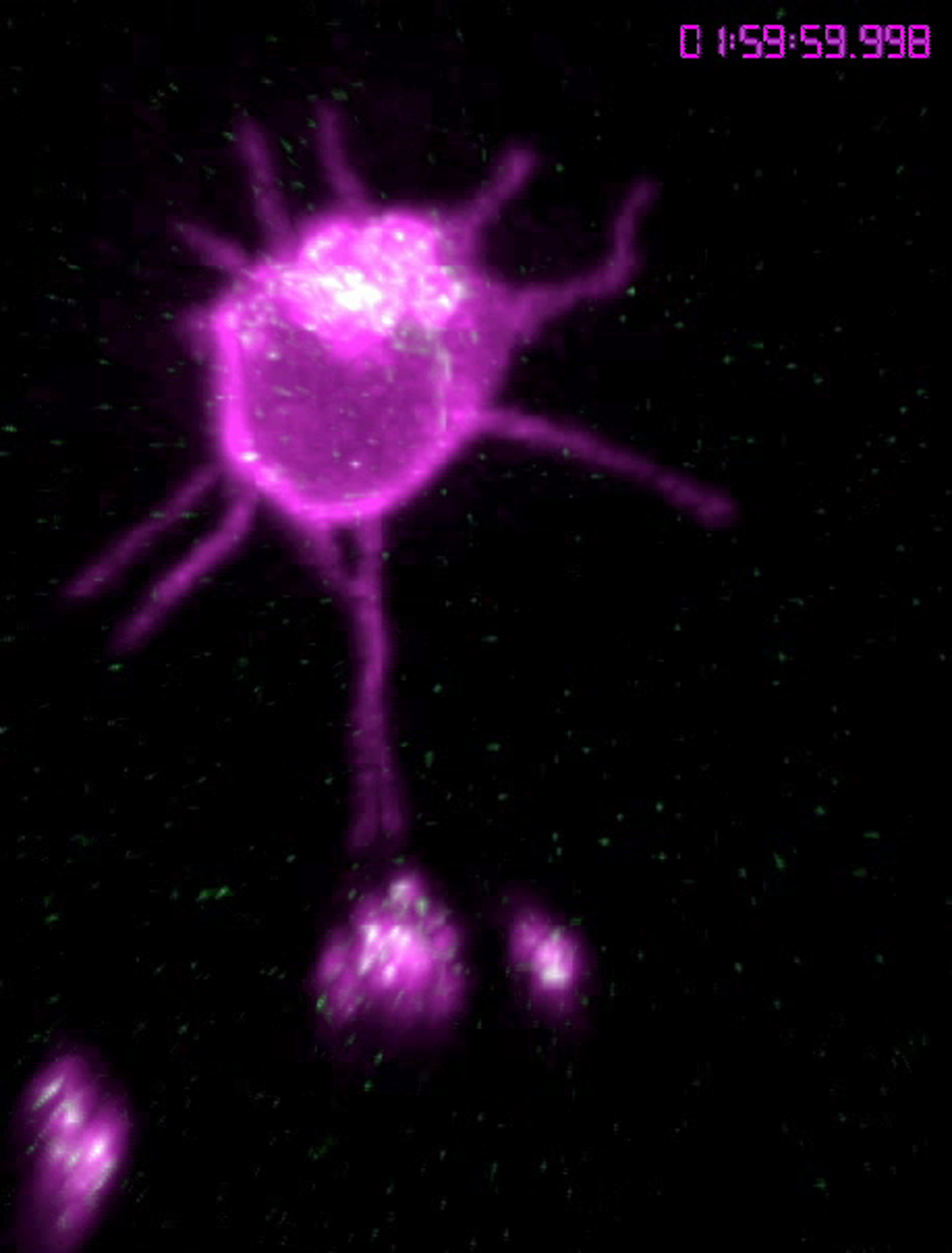

Supplement: Movie S13. Contact with Lam1 Directs Specific Kif5c560-YFP Accumulation at the Tip of a Neurite In Vitro (Part 1) — Retinas from ath5:GAP-RFP (magenta) embryos injected with Kif5c560-YFP RNA (green) at the one-cell stage were dissociated and plated on poly-L-lysine with scattered Lam1-coated 1 uM polystyrene beads (visible by autofluorescence, circled in white at t = 0:00). Contact of a neurite with a clump of Lam1 beads induces the translocation of YFP signal to the contact point. Time is shown in hr:min:s:ms. [file mmc13.jpg]

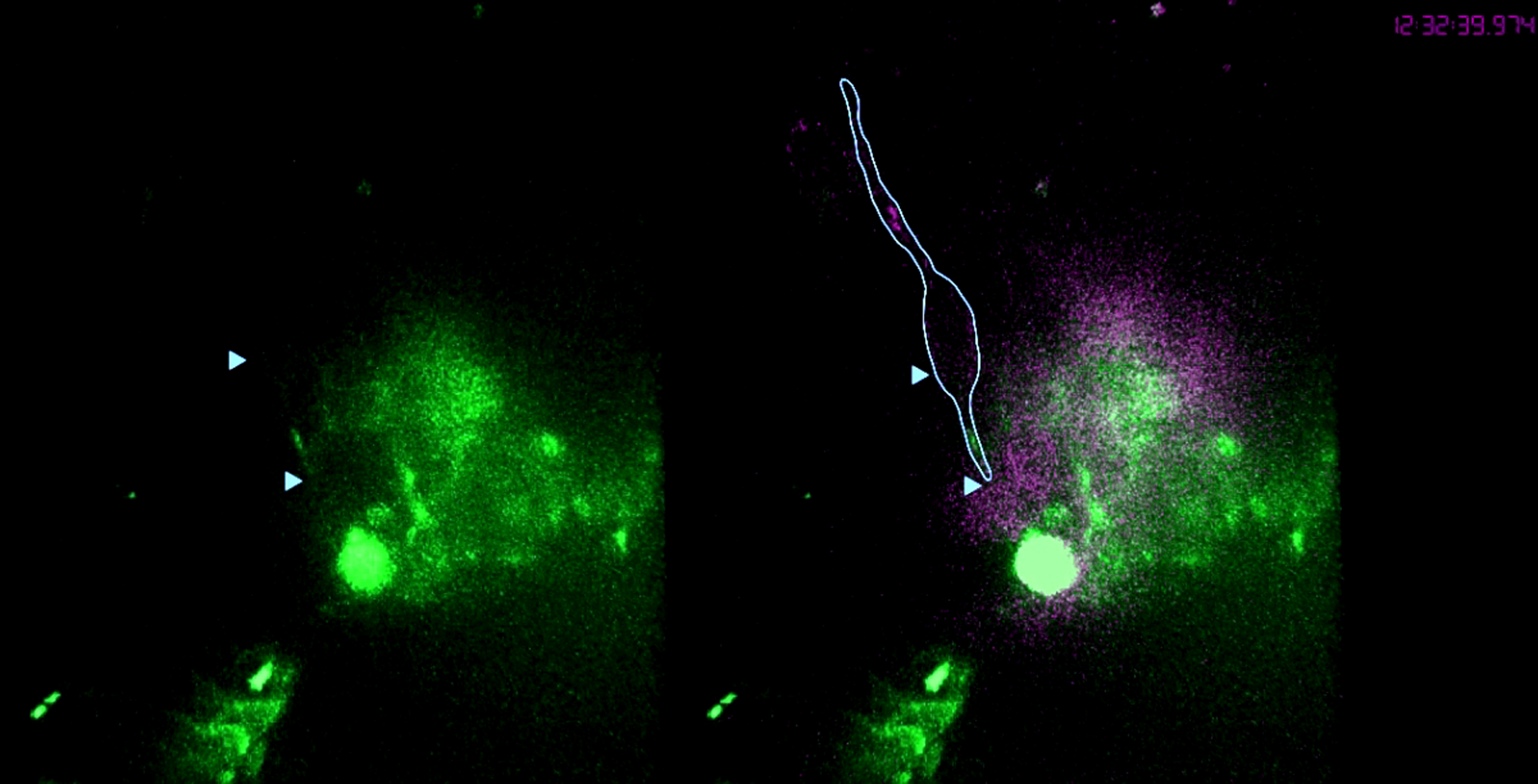

Supplement: Movie S16. Lam1 Contact Directs Stable and Specific Kif5c560-YFP Accumulation in the Contacting Neurite Followed by Axon Extension In Vivo — Mosaic embryos with WT ath5:GAP-RFP-labeled (magenta channel), Kif5c560-YFP-expressing (green channel) RGCs within a lamα1 morphant environment were implanted with Lam1-coated beads at ∼24 hpf and imaged by time-lapse confocal microscopy beginning at ∼35 hpf. As ath5:GAP:RFP signal begins to increase, this cell exhibits Stage 2 behavior with oscillating Kif5c560-YFP accumulations (double arrowheads). This cell then extends a neurite that comes in contact with the Lam1-coated bead (single arrowhead, bead marked “B” at t = 0:00), causing the stabilization of this process and specific and stable Kif5c560-YFP accumulation. This process then extends to form the axon. Time is shown in hr:min:s:ms. [file mmc16.jpg]
